# Roads as a contributor to landscape-scale variation in bird communities

**DOI:** 10.1038/s41467-020-16899-x

**Published:** 2020-07-07

**Authors:** Sophia C. Cooke, Andrew Balmford, Paul F. Donald, Stuart E. Newson, Alison Johnston

**Affiliations:** 1 Department of Zoology, University of Cambridge, The David Attenborough Building, Pembroke Street, Cambridge, CB2 3QZ UK; 2 BirdLife International, The David Attenborough Building, Pembroke Street, Cambridge, CB2 3QZ UK; 3 British Trust for Ornithology, The Nunnery, Thetford, Norfolk, IP24 2PU UK; 4 Cornell Lab of Ornithology, Cornell University, 159 Sapsucker Woods Road, Ithaca, NY 14850 USA

**Keywords:** Biodiversity, Conservation biology, Ecological modelling, Urban ecology

## Abstract

Roads and their traffic can affect wildlife over large areas and, in regions with dense road networks, may influence a high proportion of the ecological landscape. We assess the abundance of 75 bird species in relation to roads across Great Britain. Of these, 77% vary significantly in abundance with increasing road exposure, just over half negatively so. The effect distances of these negative associations average 700 m from a road, covering over 70% of Great Britain and over 40% of the total area of terrestrial protected sites. Species with smaller national populations generally have lower relative abundance with increasing road exposure, whereas the opposite is true for more common species. Smaller-bodied and migratory species are also more negatively associated with road exposure. By creating environmental conditions that benefit generally common species at the expense of others, road networks may echo other anthropogenic disturbances in bringing about large-scale simplification of avian communities.

## Abstract (web summary)

Roads are widespread and can impact ecological communities. Cooke et al. use data for 75 bird species across Great Britain to show that common species are disproportionately abundant near roads, whereas rarer, smaller-bodied and migrant species are more likely to be negatively associated with roads.

## Introduction

The ever-expanding environmental footprint of humans is affecting global wildlife populations via a wide range of mechanisms, many of which we are only beginning to understand. Extinctions and population declines are widespread^1,2^, but not evenly spread across taxa. It has been argued that differences in species’ abilities to tolerate anthropogenic disturbance are leading to the simplification of species assemblages in human-disturbed environments^3–8^.

Known human drivers of population change are numerous and include habitat loss^9^, human–wildlife conflict^10^, overharvesting^11^ and climate change^12^. In recent years, another environmental issue has become a subject of increasing attention—the extensive and expanding global road network. Forty-five million lane-kms of paved roads traverse the Earth’s land surface^13^ serving around 1.3 billion vehicles^14^, figures that are expected to increase to 70 million lane-km^13^ and 2.8 billion vehicles^15,16^ by 2050. Yet, efforts to mitigate potential road impacts on wildlife are minimal or non-existent in most countries. Only 10% of the countries that have so far submitted a 6th National Report for the Convention on Biological Diversity (via the Clearing-House Mechanism), make any mention of roads as a threat to biodiversity^17^.

Roads are a source of noise, wildlife–vehicle collisions, chemical pollution and visual disturbance, including artificial light^18–21^. Their construction leads to fragmentation effects and changes in local habitat, and often exposes surrounding areas to further development and other human activities^22,23^. Roads have been shown to affect local populations of a range of taxa, and their impacts can extend far from the roads themselves. Studies have measured effect distances of several hundred metres, with some reporting distances of over a kilometre^21,24,25^. Birds show similar patterns to other groups, exhibiting behavioural changes, physiological responses and population changes around roads^26–31^. Many of the studies behind these findings, however, are relatively small-scale, and our understanding of the larger-scale relationships between roads and animal populations is limited^32^. In addition, while predictors of species’ involvements in vehicle collisions have been studied previously^33,34^, in general, predictors of road impacts on wildlife populations are poorly understood.

Various species characteristics have the potential to affect or predict associations between birds and roads. Communication in smaller-bodied species may be more affected by road noise, due to their typically quieter and higher-frequency songs^28,35,36^, and body mass may affect the likelihood of involvement in collisions^33,34^. Habitat generalists may be more able to adapt to disturbance by roads than specialists^37^, and therefore be more likely to utilise roadside habitat, and previous work has shown migratory populations to be reduced around roads more than resident species, possibly due to a more limited ability to adapt to noise^38,39^. In addition, species with reduced abundances around roads may also have smaller national population sizes, either because roads have contributed directly to their declines or because their national scarcity is caused by their inability to tolerate disturbance, which may also manifest itself in an avoidance of roads.

Great Britain has one of the densest road networks in the world, with over 80% of land falling within 1 km of a road. We use data from the extensive UK Breeding Bird Survey (BBS) to analyse populations of 75 British bird species in relation to the paved road network, and to assess predictors of these patterns. As potential predictors, we choose three species-level characteristics—mean body mass, migratory tendency and an index of habitat specialisation—and two population-level characteristics—national population size and long-term national population trend. By assessing patterns of bird distribution in relation to roads across the whole of Great Britain, we find evidence to suggest that roads may contribute to broad-scale simplification of avian communities. Our findings provide much-needed information for potential road mitigation and conservation around roads.

## Results

### Associations between road exposure and bird abundance

We calculated the road exposure of almost 20,000 BBS transect sections using the locations of all paved roads (as mapped in 2013) within a 5-km radius of the midpoint of each transect section. Within these calculations, we estimated the spatial scale of the relationship between distance to road and road exposure (determined by a parameter ‘*k*’) for each species separately. We calculated species-specific mean annual bird counts, across 2012–2014 inclusive, for each transect section. We then modelled the mean annual counts of 75 species in relation to road exposure, using Poisson generalised additive mixed models (GAMMs), whilst also accounting for other potential predictors of bird abundance.

**Fig. 1:**
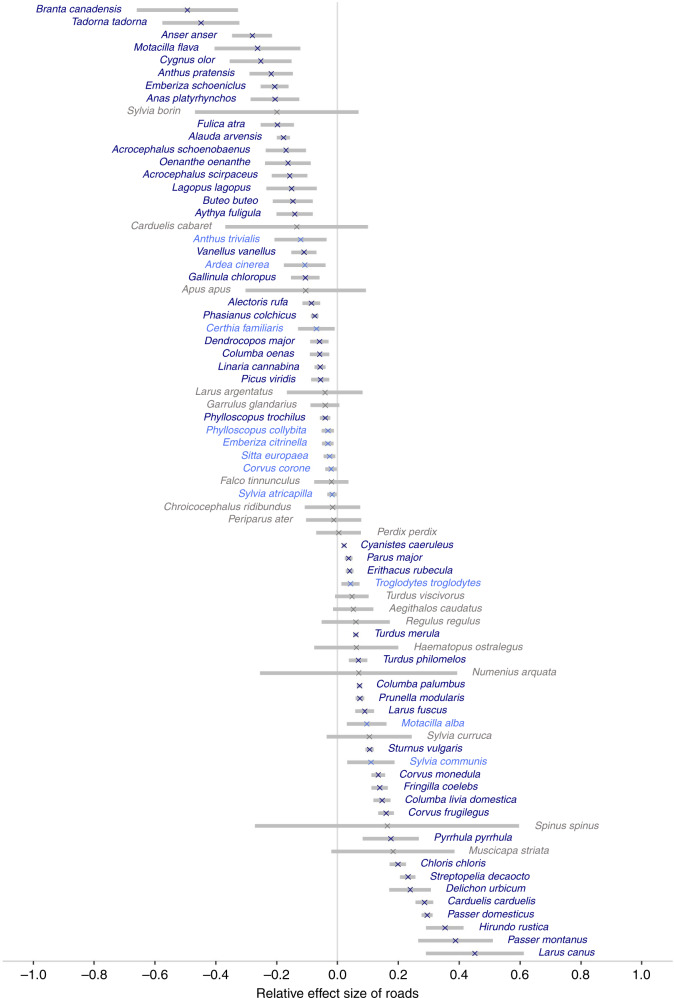
Relative effect size of associations between road exposure and bird abundance. For each species, the relative effect size was calculated as a composite of the magnitude of the effect size of road exposure and the spatial scale over which the effect could be detected (the latter being determined by the parameter ‘*k*’). Species with significant associations, determined using a critical alpha level of 0.05, are labelled in blue, with those whose significant associations were retained after Bonferroni correction in dark blue. Grey bars depict 95% confidence intervals.

Our results show the abundance of 77% (*n* = 58/75) of species tested to be significantly associated with road exposure (determined using a critical alpha level of 0.05). To account for the increased likelihood of Type I errors arising due to the testing of multiple species, we applied Bonferroni correction, after which 63% (*n* = 47/75) of associations retained statistical significance. Increased road exposure was associated with lower abundance in 25 species and higher bird abundance in 22 species (Fig. 1, Supplementary Table 1), and the maximum distances over which these negative and positive associations could be detected averaged 700 and 500 m, respectively. The results for all other model covariates are given in Supplementary Table 2.

**Fig. 2:**
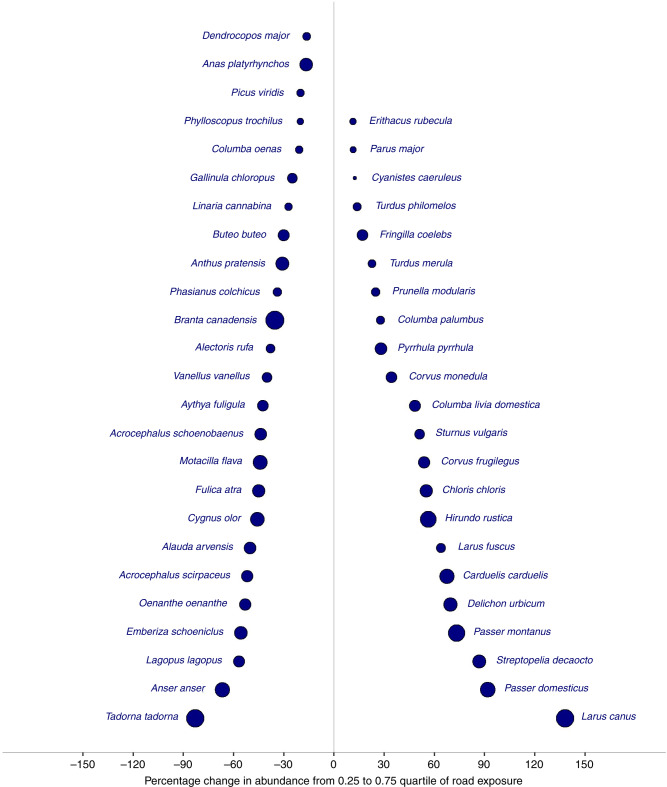
Abundance changes across the interquartile ranges of road exposure recorded for each species. Only species for which associations between road exposure and abundance were found to be significant after Bonferroni correction are featured here. The relative effect size of roads (as shown in Fig. 1) is represented by point size. Percentage change in abundance across the interquartile range of road exposure and the relative effect size are not strongly correlated as the former is affected both by the absolute numbers of birds and the range of road exposure present across counts of each species.

To estimate the real-world magnitude of the associations between road exposure and bird abundance, we used our models to predict changes in abundance across the ranges of road exposure values recorded for each species. For species with strongly significant associations between abundance and road exposure (i.e., those significant after Bonferroni correction), the mean change in abundance from the 0.25 to 0.75 quartiles of road exposure was −40% for species with negative associations, and +48% for species with positive associations (Fig. 2, Supplementary Fig. 1).

### Two species considered in detail

**Fig. 3:**
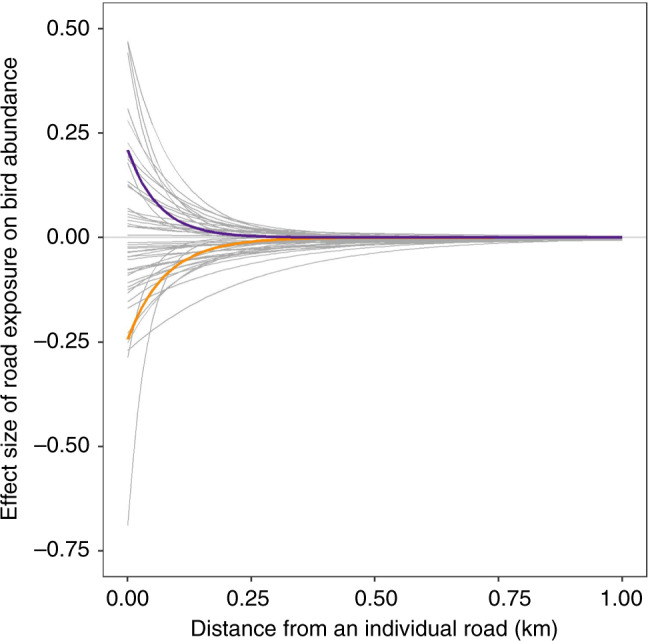
Effect curves for each species with distance from an individual road. The intercept is determined by the coefficient and the rate of decline is determined by the parameter ‘*k*’, which defines the spatial scale of the relationship between distance from road and road exposure for each species. Only species with strongly significant associations (determined with Bonferroni correction) between road exposure and bird abundance are featured here. The effect curves for Eurasian bullfinch and meadow pipit are highlighted in purple and orange, respectively.

**Fig. 4:**
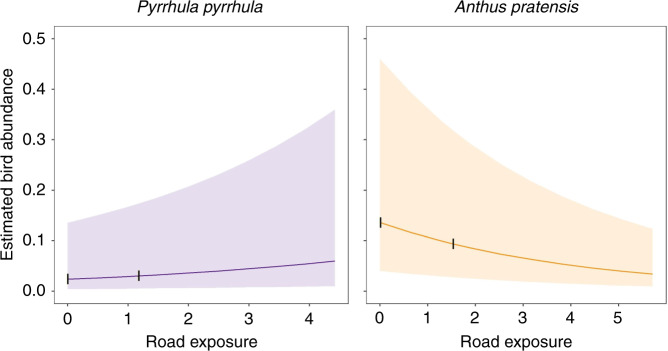
Estimated abundance of two species across the full range of road exposure recorded for each. Bird abundance refers to the number of birds within 100 m of a 200-m BBS transect section. The 0.25 and 0.75 quartiles of road exposure for each species are indicated by the vertical lines, and 95% prediction intervals by the shaded areas. These graphs are available for all species in Supplementary Fig. 1.

To explain our results in more detail, we use the examples of Eurasian bullfinch *Pyrrhula pyrrhula* and meadow pipit *Anthus pratensis*, species with significant positive and negative associations with road exposure, respectively. Eurasian bullfinch had a road exposure effect size of 0.21. This is the effect size where road exposure = 1, i.e., directly beside a single road (higher values of road exposure result from the cumulative effect of multiple roads). We would therefore expect Eurasian bullfinch abundance to be 23% (exp(0.21)) higher next to a road than in an area where road exposure = 0. This effect size declines with distance, becoming negligible at 290 m from a road (determined by the parameter ‘*k*’ and defined as the distance at which road exposure reaches <0.01, Fig. 3). Conversely, meadow pipit had a road exposure effect size of −0.24, so we predict its abundance to be 21% (1 − exp(−0.24)) lower next to a road, compared with a location with no road exposure. The maximum effect distance for meadow pipit was 350 m. These values translate to Eurasian bullfinch experiencing a 28% increase in abundance, and meadow pipit a 31% decrease in abundance, over their interquartile ranges of road exposure (Fig. 4, Supplementary Fig. 1).

### Separate analyses of major and minor roads

**Fig. 5:**
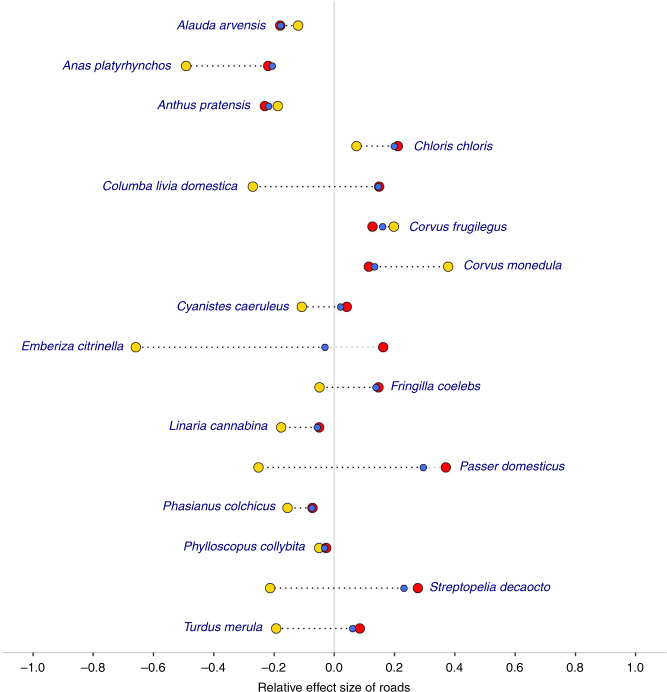
Relative effect size of associations between bird abundance and exposure to different road types. As in Fig. 1, the relative effect size was calculated as a composite of the magnitude of the effect size of road exposure and the spatial scale over which the effect could be detected. Associations with major roads are shown in yellow, minor roads in red and both road types together in blue. Only species with significant associations for all three road categories, determined using a critical alpha level of 0.05 without Bonferroni correction, are featured here.

Previous studies have suggested differences in the potential impacts of higher and lower traffic-level roads^27,40,41^. To investigate this, we analysed a subset of 29 species with high sample sizes and significant associations with road exposure (without Bonferroni correction) in relation to major roads (motorways and A-roads; mean daily traffic volume in 2013 of 17,400 vehicles^42^) and minor roads (B-, C- and D-roads; mean daily traffic volume in 2013 of 1300 vehicles^42^) separately. Of these, 16 had significant associations with both major and minor roads (Fig. 5). From our results, we can see that the original associations with roads are heavily driven by minor roads, which is as expected, given their considerably higher prevalence (87.3% of total road length^43^). Most species (13/16) were negatively associated with major roads and, of these, 7 were positively associated with minor roads. Clear exceptions were the two corvid species, rook *Corvus frugilegus* and Eurasian jackdaw *Corvus monedula*, both of which were positively associated with minor roads, and even more so with major roads. The full results for this analysis are presented in Supplementary Table 3, and effect curves for all three road categories are compared for each species in Supplementary Fig. 2.

### Species characteristics and associations with road exposure

To assess predictors of the associations we found between road exposure and bird abundance, we analysed the relative effect sizes (of all roads together) in relation to five species characteristics: mean body mass, migratory tendency, an index of habitat specialisation, national population size and long-term national population trend, using a generalised estimating equation. Within this, we accounted for non-independence resulting from similarity within phylogenetic families. We also weighted each species by 1/variance of the effect size of road exposure, to increase the influence of species with more precise association estimates between bird abundance and road exposure.

**Fig. 6:**
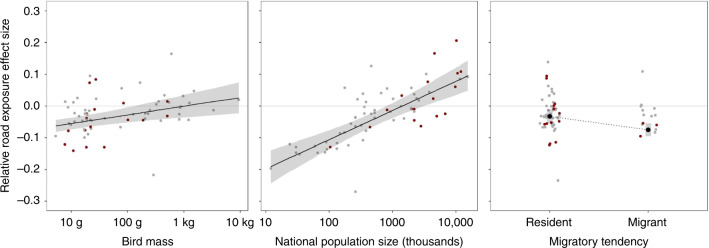
Relationships between species characteristics and associations with road exposure. Black lines/points represent the relationships between relative effect size and each characteristic, from a model in which all five characteristics were included. In all, 95% prediction intervals around each relationship are shown by the shaded grey bars. The grey and red points represent the sum of the predicted effect size and the model residual for each species—those in red are in the top 25% of model weight and thus had the strongest influence on the model.

We found that species with smaller national population sizes had generally  lower abundance with increasing road exposure, whereas the opposite was true for more common species (Table 1, Fig. 6). We also found migrants and smaller-bodied species to be more negatively associated with road exposure than resident and larger-bodied species. No variables included in the models had variance inflation factors greater than 2.0, indicating that multicollinearity among the predictors was low and unlikely to affect the results. We found no significant links between the relative effect size of road exposure and habitat specialisation or long-term national population trend.

**Table 1:** Relationships between species characteristics and associations with road exposure.

Characteristic	Effect size	Standard error	*P* value
Mean body mass	0.027	0.009	0.004
Migratory tendency	−0.042	0.012	<0.001
Habitat specialisation	0.08	0.10	0.43
National population size	0.092	0.018	<0.001
Long-term national population trend	0.012	0.061	0.84

## Discussion

Our study provides insights into broad-scale associations between paved road exposure and local bird abundance, and considers interspecific variation in these associations in relation to species characteristics. Of the 75 species we tested, 63% showed strongly significant variation in abundance with increasing road exposure, with 53% of these exhibiting reduced abundance. When major and minor roads were analysed separately, of the species with significant associations with major roads, 81% were negative. Finally, we found the effect sizes of road exposure to be more negative for rarer, smaller-bodied and migrant species.

Several smaller-scale studies have shown bird abundance to increase or decrease with proximity to roads^26,41,44,45^ with similar scales of change and mean effect distances to those found here^24,44,45^. Reductions in abundance may be attributed to direct mortality from collisions^19^, or avoidance of areas around roads due to noise^46,47^ or visual disturbance^18,30,48,49^, which decrease the perceived habitat quality. This can lead not only to population reductions but also to changes in population structures^50,51^. Increases in abundance could be explained by attraction to the road surface for food, grit or heat^19,52,53^, or to roadside habitat^54,55^ and associated structures such as powerlines and fences^56^.

The influence of roadside habitat is particularly difficult to quantify here as, although we incorporated habitat in our models, it was not captured at high enough resolution to account for subtle changes in roadside areas. Roads can create a variety of edge habitat^55^, which may be of benefit to some species but be avoided by others. Britain has very few areas of lowland semi-natural habitat, and so road verges, which often contain hedgerows and trees, may be important for some species. In addition, many roads may have been built alongside existing edge habitat, in which some birds were perhaps already at reduced or increased abundance. However, some previous studies have controlled for habitat and still found negative effects of road traffic, including on several species in this analysis^24,44^. Most likely, our results arise from a combination of road and habitat effects, both varying in importance around different road types. We found several species to differ in their associations with major and minor roads, with varying effect distances, which suggests that different mechanisms may be of greater or lesser importance around each. In particular, our finding of some species being associated positively with minor roads and negatively with major roads suggests that high levels of traffic may outweigh habitat benefits, even for those species that are able to tolerate lower-level disturbance.

Our finding of a significant positive relationship between national abundance and road exposure effect size could imply that rarer birds are more inclined to avoid roads. It is possible that roadside habitat is unattractive to rarer species, as their reduced national abundance is, in part, due to their reduced ability to thrive under human disturbance in general. This reduction in competition in areas of higher road exposure could then result in an increase in abundance of species that are more able to tolerate human disturbance and are therefore more common nationally. Smaller-bodied species and migrants may also be found in lower abundances around roads due to increased sensitivity to road-related disturbances such as noise.

As we did not find a significant link between abundance around roads and long-term national population trend, the broader outcome of this lower abundance of some species around roads is difficult to interpret. It could be that road areas act as a sink for these species, or that they are simply avoided by them, but that abundance in areas with lower road exposure has increased enough to stabilise the national population. However, it is important to note that our measures of long-term population trends only began in 1970. Although traffic volume in Great Britain has increased greatly in that time, the total road length has increased by less than 25%^42^. Therefore, by the beginning of this period, sensitive species may have already adjusted to the presence of the road network.

Shifts in species assemblages in areas of high human disturbance have been identified in both urban^4,5^ and agricultural^57^ environments, and in response to climate change^5,6^. Rather than declines of so-called ‘loser’ species happening in isolation, simultaneous replacement of those species by expanding ‘winner’ species occurs^3,7,8,58^. These processes, it is suggested, are leading to homogenisation, or simplification, of biodiversity in large areas. Our results indicate that roads may create environments that benefit already common species at the expense of others. In this way, they may contribute to this simplification effect, maintaining total bird numbers but reducing species richness and diversity. Given the extent of the global road network, it is likely that our findings are not unique to Britain, and so studies to test this pattern in other countries would be beneficial. Replicability of this study is dependent on wide-scale and high-resolution bird and road data, but with increasing citizen science projects worldwide, there may already be many areas in which this is possible. Furthermore, if changes in both road and bird densities were analysed over time, and areas monitored before and after road development, this could give a stronger idea of the level of causality between the two, and an ability to predict the impact of further construction of transport infrastructure.

**Fig. 7:**
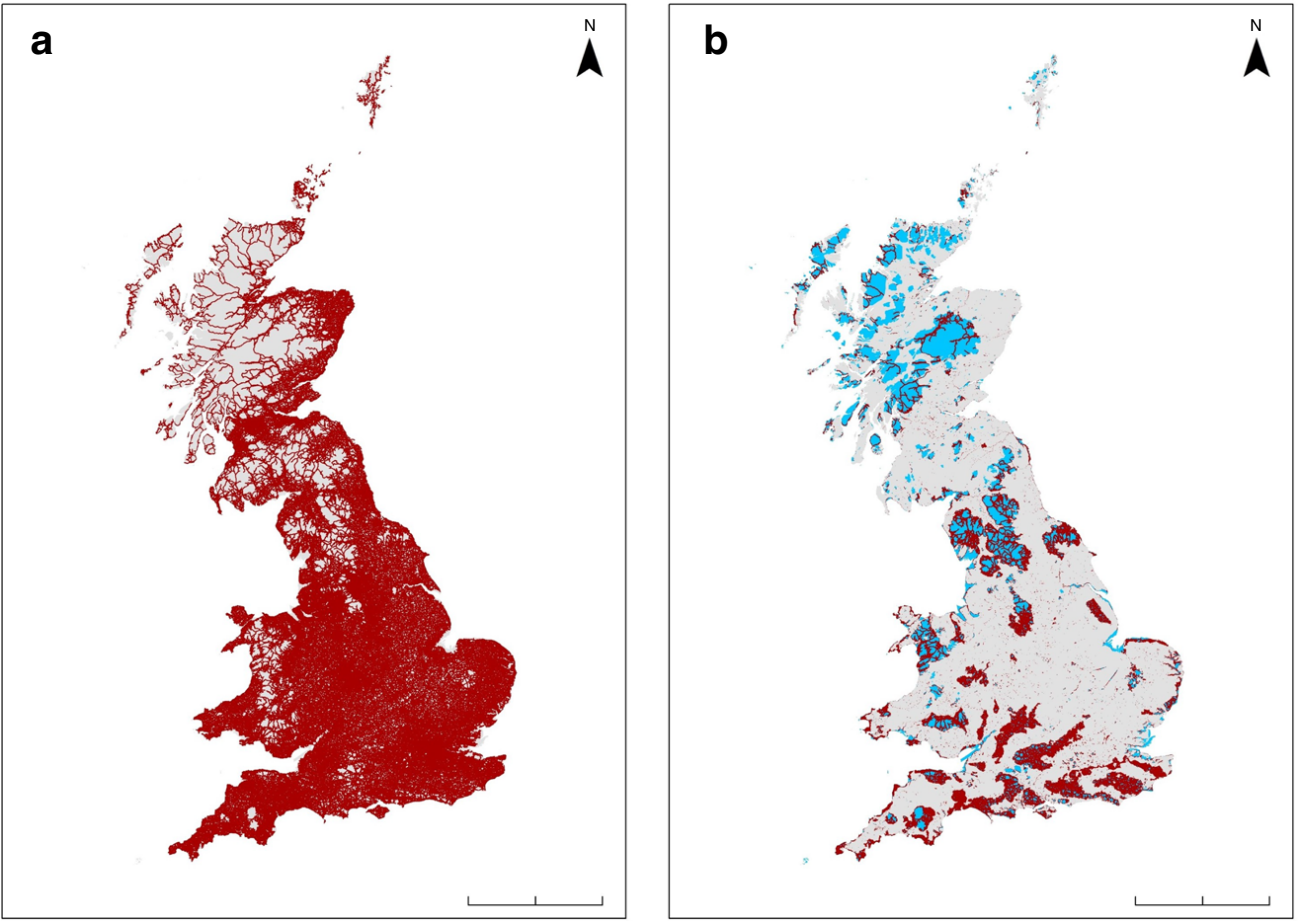
Areas of Great Britain and terrestrial protected areas within 700 m of a road. Blue represents terrestrial protected areas and red represents areas of **a** Great Britain and **b** terrestrial protected areas within the mean effect distance, 700 m, of associations between roads and bird abundance variation. Scale bars denote 200 m. Great Britain boundary shapefile obtained from ONS^74^.

Compression of already-vulnerable species into shrinking pockets of low road density may increase future declines and extinctions in countries with high road densities. Our results showed that, for species in reduced abundance with increasing road exposure, this effect extended to a mean of 700 m from a road. Almost three-quarters (72%) of Great Britain’s land surface falls within 700 m of a road (Fig. 7), leaving limited areas with road exposure low enough not to be associated with abundance changes. In addition, disturbance by roads may be a limiting factor for the success of conservation projects situated near roads. In Great Britain, 41% of the total area of terrestrial protected sites lies within 700 m of a road (Fig. 7). Further work to identify cost-effective methods of mitigation is urgently required, and a particular focus on noise reduction (of both vehicle engines and tyre-road interactions) would likely be beneficial^59^. Global traffic and road construction are predicted to continue increasing on a large scale, and so mitigation of road impacts on wildlife must be a priority for governments and land managers. As road-related disturbance such as noise pollution is thought to be harmful also to humans^60–62^, mitigation for wildlife could be approached in tandem with that for people.

## Methods

### Overview

We modelled count data from the UK BBS for 75 species in relation to the proximity of nearby roads, whilst also accounting for other potential predictors of bird abundance. In a second step, we then analysed these results with respect to a range of species-specific characteristics to identify predictors of associations between road exposure and bird abundance. We used ArcMap 10.5.1^63^ and R 3.6.0^64^ for all data preparation and analyses.

### Data collation and preparation

We obtained bird count data from the UK BBS, a nationwide survey in which experienced volunteers walk two 1-km transects across a 1-km square, each transect being divided into 200-m sections. These transects mostly do not follow roads (64% of the transect sections used in this analysis did not follow a paved road along any part of them). We extracted counts from squares that had been surveyed every year from 2012 to 2014 inclusive. We then calculated the mean bird count for each 200-m transect section across that period, removing any species with a total mean annual count <100. We also extracted the dominant habitat type recorded for each transect section. Our final dataset contained counts from 19,709 transect sections in 2033 squares. Preparation of these data is detailed in Supplementary Methods.

We obtained shapefiles for all road classes (major roads: motorways and A-roads; minor roads: B-, C- and D-roads) in Great Britain, as recorded in 2013. We then used kernel density estimation to calculate a measure of road exposure for the midpoint of every 200-m transect section, using the locations of all roads within a 5-km radius. We optimised the spatial scale of the relationship between the distance from road and road exposure, represented by the parameter *k*, for each species individually. Further detail on the preparation of the road data can be found in Supplementary Methods.

To account for factors other than road exposure that we expected to affect bird abundance, we calculated human population density, temperature and rainfall values for the midpoint of each transect section. We also calculated the following for 5-km buffers around each midpoint: tree cover density, proportion of arable land (as a proxy for yield) and the largest field area (as a proxy of agricultural intensity). For information on data sources and calculation of these data see Supplementary Methods.

### Data analysis

Our goal was to understand how bird abundance varies in relation to roads, and to identify the characteristics of species that best predict these associations. We therefore modelled counts of each species, as recorded on BBS transects, as a function of road exposure and other factors that we also expected to affect bird abundance (habitat (as recorded in the BBS), proportion of arable land, largest field area, human population density, temperature, rainfall and tree cover density). We ran Poisson GAMMs for each species separately, using the R package ‘mgcv’^65^. We fitted each variable with a linear effect on the response, but from initial inspection of the relationships between the proportion of arable land and bird count, we fitted the proportion of arable land quadratically for 11 species (Supplementary Table 1). We incorporated BBS square as a random effect (to account for the non-independence of counts at each square’s transect sections), and we included a spatial smooth to account for large-scale variation in bird abundance not associated with the other covariates. The spatial smooth included Easting and Northing as a joint tensor product smooth with a maximum of 50 degrees of freedom (selected with preliminary analyses).

We performed an additional analysis of species that showed significant associations with road exposure (without Bonferroni correction), incorporating major and minor road exposure in separate models. As there are fewer major roads, and fewer BBS squares near major roads (93% and 47% of transect sections were within 1000 and 100 m of a minor road, respectively, and 44% and 9% were within 1000 and 100 m of a major road, respectively), for this analysis, we selected species with total mean annual counts >1000, in a minimum of 100 BBS squares, and only used squares within 5 km of a major road.

Cooke et al.^66^ demonstrated the importance of accounting for differences in detectability of birds when analysing the impacts of roads, but this is only possible with large sample sizes and a broad spread of data in relation to road exposure. As here we were interested in interspecific variation in patterns and hence required a large number of species, we could not account for detectability, but confirmed through sensitivity testing on 48 more commonly recorded species that this was only likely to modify the size of significant effects slightly and not change their direction (Supplementary Methods).

To assess significance, we calculated confidence limits for each species as the effect size ± standard error multiplied by the appropriate *t* value from the Student’s *t* distribution, using a critical alpha level of 0.05. We then applied Bonferroni correction, dividing our critical alpha level by the number of species tested (*n* = 75) and recalculating the confidence limits. In both cases, we declared significance if the confidence limits did not span zero. To allow easier comparison of results between species, we calculated the relative effect size for each, dividing the effect size by the log_10_-transformed value of *k* used for that species (*k* is inversely proportional to the distance over which the effect occurred), thus combining the magnitude of the effect with the spatial area over which the effect occurred. We then used our models to predict bird abundance across the ranges of road exposure recorded for each species, while holding all other continuous covariates at the mean values of the counts of that species. For the two categorical covariates (BBS square and dominant habitat type for each 200-m transect section), we used the BBS square with the smallest absolute random effect size (closest to the average BBS square) and the habitat with the largest number of counts for that species.

To test whether species characteristics were associated with different directions and magnitudes of road exposure effects on bird abundance, we modelled the relationships between the relative effect size of road exposure and five chosen characteristics: mean body mass, migratory tendency, an index of habitat specialisation, national population size and long-term national population trend (1970–2016). We extracted mean body masses from Robinson^67^ and migratory tendency data (in categorical form—resident or migrant) from McInerny et al.^68^. We obtained an index of how specialised or generalised a species is in its habitat use from Davey et al.^6^, national population estimates for Great Britain from Musgrove et al.^69^ and long-term trend data from DEFRA^70^. We also obtained relative brain mass estimates, which we calculated from data provided in Moller and Erritzoe^71^; however, we excluded this measure from subsequent analyses due to its correlation with mean body mass, and because these data were available for fewer species. We performed the generalised estimating equation using the R package ‘zelig’^72^. Within this, we incorporated taxonomic family as a grouping factor to account for any non-independence between species resulting from phylogenetic relatedness. To increase the influence of species with more precise estimates of the effect of road exposure, we also weighted each species by 1/variance of the effect size of road exposure.

### Reporting summary

Further information on research design is available in the Nature Research Reporting Summary linked to this article.

## Supplementary information

Supplementary Information

Peer Review

Reporting Summary

## Data Availability

The data analysed in this study are available online through ‘Apollo, the University of Cambridge’s repository. 10.17863/CAM.50241’^73^.

## Code availability

The codes used in this study are available online through ‘Apollo, the University of Cambridge’s repository. 10.17863/CAM.50241’.
