# Supplementary material for: Roads as a contributor to landscape-scale variation in bird communities
Source: Nat Commun. 2020 Jul 7;11:3125. doi: 10.1038/s41467-020-16899-x (PMC7340783; doi:10.1038/s41467-020-16899-x)
Supplement: Supplementary file 1 — Supplementary Information [file 41467_2020_16899_MOESM1_ESM.pdf]

Cooke et al. Roads as a contributor to landscape-scale variation in bird communities: Supplementary Information

Supplementary Tables

Supplementary Table 1. GAMM parameters and road exposure results for each species. The threshold significance level of 0.0007 was determined with Bonferroni correction.

**Scientific name**	**Common name**	**Survey visit**	**Prop. arable land fit**	***k***	**Road exposure effect size**	**Significance level**
Acrocephalus schoenobaenus	Sedge warbler	Late	Linear	5	-0.119	$P < 0.0007$
Acrocephalus scirpaceus	Reed warbler	Late	Linear	3	-0.075	$P < 0.0007$
Aegithalos caudatus	Long-tailed tit	Early	Linear	17	0.063	NS
Alauda arvensis	Eurasian skylark	Early	Quadratic	4	-0.107	$P < 0.0007$
Alectoris rufa	Red-legged partridge	Early	Quadratic	2	-0.026	$P < 0.0007$
Anas platyrhynchos	Mallard	Early	Linear	25	-0.288	$P < 0.0007$
Anser anser	Greylag goose	Early	Linear	4	-0.169	$P < 0.0007$
Anthus pratensis	Meadow pipit	Early	Linear	13	-0.243	$P < 0.0007$
Anthus trivialis	Tree pipit	Late	Linear	3	-0.058	<0.05
Apus apus	Common swift	Late	Linear	20	-0.136	NS
Ardea cinerea	Grey heron	Early	Linear	5	-0.075	$P < 0.05$
Aythya fuligula	Tufted duck	Early	Linear	4	-0.085	$P < 0.0007$
Branta canadensis	Canada goose	Early	Linear	25	-0.690	$P < 0.0007$
Buteo buteo	Common buzzard	Early	Linear	8	-0.133	$P < 0.0007$
Carduelis cabaret	Lesser redpoll	Early	Linear	14	-0.154	NS
Carduelis carduelis	European goldfinch	Early	Linear	12	0.308	$P < 0.0007$
Certhia familiaris	Eurasian treecreeper	Early	Linear	6	-0.054	$P < 0.05$
Chloris chloris	European greenfinch	Early	Linear	9	0.189	$P < 0.0007$
Chroicocephalus ridibundus	Black-headed gull	Early	Linear	9	-0.016	NS
Columba livia domestica	Feral pigeon	Early	Linear	7	0.123	$P < 0.0007$
Columba oenas	Stock dove	Early	Quadratic	4	-0.036	$P < 0.0007$
Columba palumbus	Common woodpigeon	Early	Linear	5	0.050	$P < 0.0007$
Corvus corone	Carrion crow	Early	Linear	8	-0.019	$P < 0.05$
Corvus frugilegus	Rook	Early	Linear	7	0.135	$P < 0.0007$
Corvus monedula	Eurasian jackdaw	Early	Linear	9	0.128	$P < 0.0007$
Cyanistes caeruleus	Blue tit	Early	Linear	2	0.006	$P < 0.0007$
Cygnus olor	Mute swan	Early	Linear	8	-0.228	$P < 0.0007$
Delichon urbicum	Common house martin	Late	Linear	9	0.228	$P < 0.0007$
Dendrocopos major	Great spotted woodpecker	Early	Linear	6	-0.046	$P < 0.0007$
Emberiza citrinella	Yellowhammer	Early	Quadratic	2	-0.010	$P < 0.05$
Emberiza schoeniclus	Common reed bunting	Early	Linear	4	-0.125	$P < 0.0007$
Erithacus rubecula	European robin	Early	Linear	7	0.033	$P < 0.0007$
Falco tinnunculus	Common kestrel	Early	Quadratic	4	-0.012	NS
Fringilla coelebs	Common chaffinch	Early	Linear	20	0.180	$P < 0.0007$
Fulica atra	Eurasian coot	Early	Linear	6	-0.154	$P < 0.0007$
Gallinula chloropus	Common moorhen	Early	Linear	7	-0.090	$P < 0.0007$
Garrulus glandarius	Eurasian jay	Early	Linear	8	-0.037	NS

Haematopus ostralegus	Eurasian oystercatcher	Early	Linear	12	0.066	NS
Hirundo rustica	Barn swallow	Late	Linear	18	0.443	$P < 0.0007$
Lagopus lagopus	Red grouse	Early	Linear	2	-0.046	$P < 0.0007$
Larus argentatus	Herring gull	Early	Linear	35	-0.064	NS
Larus canus	Common gull	Early	Linear	11	0.470	$P < 0.0007$
Larus fuscus	Lesser black-backed gull	Early	Linear	2	0.027	$P < 0.0007$
Linaria cannabina	Common linnet	Early	Quadratic	2	-0.017	$P < 0.0007$
Motacilla alba	Pied/white wagtail	Early	Linear	14	0.110	$P < 0.05$
Motacilla flava	Yellow wagtail	Late	Quadratic	9	-0.251	$P < 0.0007$
Muscicapa striata	Spotted flycatcher	Late	Linear	18	0.228	NS
Numenius arquata	Eurasian curlew	Early	Linear	35	0.107	NS
Oenanthe oenanthe	Northern wheatear	Late	Linear	3	-0.078	$P < 0.0007$
Parus major	Great tit	Early	Linear	6	0.028	$P < 0.0007$
Passer domesticus	House sparrow	Early	Linear	9	0.281	$P < 0.0007$
Passer montanus	Tree sparrow	Early	Quadratic	16	0.467	$P < 0.0007$
Perdix perdix	Grey partridge	Early	Quadratic	4	0.002	NS
Periparus ater	Coal tit	Early	Linear	20	-0.016	NS
Phasianus colchicus	Ring-necked pheasant	Early	Linear	2	-0.023	$P < 0.0007$
Phylloscopus collybita	Common chiffchaff	Late	Linear	6	-0.025	$P < 0.05$
Phylloscopus trochilus	Willow warbler	Late	Linear	2	-0.012	$P < 0.0007$
Picus viridis	European green woodpecker	Early	Linear	4	-0.034	$P < 0.0007$
Prunella modularis	Dunnock	Early	Linear	6	0.057	$P < 0.0007$
Pyrrhula pyrrhula	Eurasian bullfinch	Early	Linear	16	0.210	$P < 0.0007$
Regulus regulus	Goldcrest	Early	Linear	25	0.083	NS
Sitta europaea	Eurasian nuthatch	Early	Linear	2	-0.008	$P < 0.05$
Spinus spinus	Eurasian siskin	Early	Linear	90	0.319	NS
Streptopelia decaocto	Eurasian collared dove	Early	Linear	7	0.195	$P < 0.0007$
Sturnus vulgaris	Common starling	Early	Linear	4	0.063	$P < 0.0007$
Sylvia atricapilla	Eurasian blackcap	Late	Linear	5	-0.013	$P < 0.05$
Sylvia borin	Garden warbler	Late	Linear	50	-0.339	NS
Sylvia communis	Common whitethroat	Late	Quadratic	35	0.169	$P < 0.05$
Sylvia curruca	Lesser whitethroat	Late	Quadratic	14	0.119	NS
Tadorna tadorna	Common shelduck	Early	Linear	4	-0.270	$P < 0.0007$
Troglodytes troglodytes	Eurasian wren	Early	Linear	20	0.055	$P < 0.05$
Turdus merula	Common blackbird	Early	Linear	5	0.042	$P < 0.0007$
Turdus philomelos	Song thrush	Early	Linear	11	0.071	$P < 0.0007$
Turdus viscivorus	Mistle thrush	Early	Linear	10	0.047	NS
Vanellus vanellus	Northern lapwing	Early	Linear	3	-0.053	$P < 0.0007$

Supplementary Table 2. Significance (determined without Bonferroni correction) and coefficients for all other covariates included in the GAMM for each species. The reference habitat category for all species is woodland. Effect sizes for the continuous covariates are not directly comparable as they were not standardised.

	Habitat								Prop. arable land	Prop. arable land^2	Largest cropping unit	Human population density (log_{10})	Temperature	Rainfall	Tree cover density
Scientific name	Scrubland	Semi-natural grassland and marsh	Heathland and bogs	Farmland	Human sites	Water-bodies (freshwater)	Coastal	Inland rock							
Acrocephalus schoenobaenus	*1.752	*2.138	0.806	*1.253	*0.882	*3.206	*1.498	-23.76	*1.561	-	*0.709	0.053	-0.108	*3.611	*1.627
Acrocephalus scirpaceus	*1.45	*2.002	-24.5	*0.661	0.367	*3.3	0.32	-25.16	-0.949	-	0.493	0.153	*-13.53	-4.393	*1.753
Aegithalos caudatus	0.105	*-1.219	*-1.847	*-0.614	*-0.493	0.043	*-1.149	-1.976	-0.084	-	0.197	-0.001	*4.327	*2.186	0.264
Alauda arvensis	*0.848	*2.117	*1.885	*1.567	*0.461	*0.366	*1.208	*2.105	*2.907	-0.978	-0.132	-0.058	-0.006	0.171	0.008
Alectoris rufa	-0.035	-0.373	0.323	*0.978	0.143	0.2	0.887	0.479	*5.031	*-4.137	0.152	*-0.191	-1.854	*-2.737	0.207
Anas platyrhynchos	-0.251	0.121	*-0.586	0.11	*0.257	*1.982	*0.648	-0.881	-0.172	-	-0.09	0.027	*-3.501	-1.177	-0.033
Anser anser	-0.176	0.166	*0.858	0.156	*0.846	*1.827	0.731	-26.73	0.669	-	-0.609	*0.377	8.862	1.164	0.782
Anthus pratensis	*1.24	*2.394	*2.343	*1.375	-0.182	*1.783	*1.933	*2.221	*-3.111	-	-0.214	*-0.165	-2.758	-1.472	-0.345
Anthus trivialis	*0.977	-0.231	0.286	*-1.28	*-1.817	-0.828	-1.087	0.408	*-5.441	-	0.97	*-0.469	*10.213	0.071	*1.925
Apus apus	*1.17	-0.74	0.861	-0.16	*0.615	*0.877	-1.799	0.585	1.117	-	-0.316	*0.448	2.978	2.975	-0.217
Ardea cinerea	0.095	0.106	-0.55	-0.227	0.406	*2.478	1.038	-19.58	-0.417	-	-0.124	0.212	-2.038	-1.104	-0.323
Aythya fuligula	*1.498	0.4	-1.488	-0.362	*0.568	*3.389	0.396	-27.95	0.198	-	0.02	*0.388	-0.478	-2.349	0.195
canadensis	-0.151	*0.659	0.418	0.201	0.172	*2.366	-0.239	-23.28	-0.735	-	*-0.751	-0.019	-6.801	-2.145	0.346
Buteo buteo	-0.400	*-1.085	*-1.14	*-0.298	*-1.031	-0.471	-1.751	-0.322	*0.92	-	-0.024	*-0.184	-3.072	-0.447	0.033
Carduelis cabaret	*0.662	*-1.231	-0.595	*-0.944	-0.632	-1.333	-25.14	-0.287	-0.553	-	*-2.167	-0.169	0.089	2.497	*1.298
Carduelis carduelis	*0.469	*-0.333	*-1.206	*0.501	*1.016	*0.671	-0.535	-0.953	*0.703	-	-0.001	*-0.074	*4.258	0.967	*0.388
Certhia familiaris	*-1.749	*-2.067	*-2.844	*-1.548	*-1.652	*-1.093	-18.9	-18.87	-0.25	-	-0.03	*-0.201	0.39	-0.322	0.671
Chloris chloris	*0.633	*-0.35	*-0.47	*0.347	*1.074	*0.725	-0.453	-0.244	*0.798	-	0.185	0.058	-0.057	-0.213	*0.544
Chroicocephalus ridibundus	0.314	0.353	0.581	*1.909	*1.684	*2.073	*3.395	1.404	*-2.03	-	0.154	*-0.273	1.372	-0.228	1.032
Columba livia domestica	*1.034	0.131	-1.39	*1.27	*1.987	*2.141	*1.687	-18.02	-0.693	-	-0.331	*0.41	-1.185	0.45	0.502
Columba oenas	-0.310	*-0.505	*-1.909	-0.149	-0.186	-0.289	-0.952	-1.029	*1.742	*-1.765	-0.22	0.016	-2.04	-0.702	-0.117
Columba palumbus	*-0.222	*-0.798	*-1.243	*-0.454	-0.038	*-0.173	*-0.558	*-0.859	*1.207	-	0.108	*0.094	-1.401	*0.891	*0.48
Corvus corone	-0.129	*-0.364	*-0.864	*0.173	*0.16	*0.165	-0.119	0	*-0.406	-	0.01	*0.101	-0.661	0.439	-0.037
Corvus frugilegus	*-2.119	*-0.704	*-3.4	*-0.256	*-0.223	*-0.313	*-1.634	-1.313	*1.603	-	0.079	*-0.13	*-8.462	-2.288	*-2.107
Corvus monedula	*-0.683	*-0.415	*-1.716	*0.161	*0.694	*0.213	-0.284	*0.491	0.24	-	-0.048	-0.04	-0.08	-0.33	-0.414
Cyanistes caeruleus	*-0.282	*-1.248	*-1.768	*-0.489	*-0.237	*-0.236	*-1.147	*-1.623	0.094	-	0.066	-0.013	0.672	0.048	0.098
Cygnus olor	*0.986	*1.428	-21.69	0.303	*0.914	*3.267	*2.198	-21.86	-0.288	-	0.511	*0.286	-6.139	-0.719	*-1.632
Delichon urbicum	*1.268	-0.039	-0.693	*0.935	*1.86	*0.813	*1.558	-24.19	*1.724	-	-0.318	-0.139	-3.444	-1.238	0.513
Dendrocopos major	*-0.466	*-1.937	*-1.804	*-1.013	*-0.836	*-0.894	*-1.786	*-2.193	0.01	-	*-0.337	-0.076	0.3	0.05	0.26
Emberiza citrinella	*1.321	*0.933	*0.83	*1.35	-0.015	-0.123	-1.46	1.142	*12.048	*-9.849	0.043	*-0.19	2.356	*2.379	-0.093
Emberiza schoeniclus	*1.64	*2.363	*1.552	*1.406	*0.594	*2.893	*1.775	*1.837	*1.181	-	*0.564	0.08	-0.357	*2.359	0.357
rubecula	*-0.256	*-1.337	*-1.603	*-0.595	*-0.364	*-0.349	*-1.831	*-1.046	*-0.282	-	0.036	*0.04	0.422	*0.64	0.115
Falco tinnunculus	0.518	*1.044	0.061	*0.471	0.052	0.589	-14.52	*2.09	*3.555	*-2.854	0.061	-0.072	1.885	2.285	0.314

Fringilla coelebs	*-0.124	*-1.048	*-1.367	*-0.253	*-0.211	*-0.178	*-1.338	*-1.193	*0.523	-	-0.029	*-0.144	0.042	0.096	0.178
Fulica atra	0.360	0.345	*-1.575	*-0.518	*0.418	*2.62	*1.408	-24.31	-0.148	-	0.168	*0.239	-4.349	-0.332	-0.046
Gallinula chloropus	-0.144	-0.015	*-1.148	0.023	*0.501	*2.432	-0.241	-18.43	0.027	-	-0.004	*0.119	-1.752	-0.142	-0.241
Garrulus glandarius	-0.264	*-1.572	*-1.58	*-1.243	*-1.134	*-1.084	-17.38	-1.053	*-0.536	-	-0.27	0.078	0.108	-0.061	*0.597
Haematopus ostralegus	*1.481	*1.38	*1.141	*1.711	*1.738	*2.767	*3.371	1.398	1.262	-	-1.214	-0.197	0.113	-3.106	*1.616
Hirundo rustica	*0.446	0.274	*-1.085	*1.084	*1.197	*0.804	0.716	0.585	*0.637	-	-0.327	*-0.305	-2.289	-0.238	-0.361
Lagopus lagopus	0.578	*1.036	*2.066	*-1.47	-17.52	-0.686	-16.07	*1.28	*-6.36	-	-3.034	-0.045	-0.636	-1.298	*-3.94
Larus argentatus	0.094	*1.384	-0.265	*1.582	*2.229	*1.794	*3.577	1.709	-1.08	-	*1.13	*0.299	8.124	0.88	0.606
Larus canus	0.887	1.73	1.045	*1.874	-0.24	1.112	-31.88	-28.55	*4.026	-	*-4.592	*0.859	10.869	2.091	-3.273
Larus fuscus	*2.095	*1.332	*2.446	*2.429	*2.388	*3.316	*4.038	-24.37	-0.444	-	0.027	-0.052	5.665	-1.49	0.485
Linaria cannabina	*1.505	*1.631	*1.447	*1.573	*1.153	*1.624	*1.933	*1.513	*4.517	*-3.002	0.017	-0.065	-1.633	1.006	*0.987
Motacilla alba	-0.096	-0.125	*-0.494	*0.912	*1.047	*0.871	*1.219	0.452	*-0.662	-	-0.142	*-0.227	2.538	0.534	-0.249
Motacilla flava	0.805	*2.208	-17.81	*2.234	0.455	0.901	-20.42	-18.14	*9.087	*-4.467	0.426	-0.085	-6.317	-4.762	*2.764
Muscicapa striata	*-1.029	*-1.406	*-1.51	*-0.947	-0.47	*-2.224	-19.56	-20	-0.158	-	-0.847	*-0.503	4.149	-1.015	-0.088
Numenius arquata	1.083	*1.823	*1.858	*1.38	-0.326	0.124	*3.394	*1.727	*-2.388	-	-0.999	*-0.221	1.139	-2.161	0.291
Oenanthe oenanthe	0.753	*2.359	*1.57	*1.342	1.075	*1.949	*2.553	*2.403	*-3.973	-	0.572	*0.298	-0.101	-2.146	*-4.151
Parus major	*-0.307	*-1.314	*-1.933	*-0.474	*-0.409	*-0.308	*-1.636	*-0.821	-0.124	-	0.047	0.017	*1.684	0.707	-0.017
Passer domesticus	*0.879	*-0.454	*-1.101	*1.024	*2.156	*0.997	*1.049	-0.628	*0.839	-	-0.038	*0.241	-0.855	0.474	0.454
Passer montanus	0.889	-0.027	-0.298	*1.284	*1.866	*0.955	-21.88	-20.96	*11.424	*-9.338	-0.241	*-0.28	1.672	*3.776	0.05
Perdix perdix	1.157	1.013	0.377	*1.529	-0.493	1.021	1.498	-18.67	*5.393	-2.808	-0.014	-0.152	0.828	-3.432	0.631
Periparus ater	*-0.803	*-2.823	*-2.048	*-1.674	*-0.993	*-1.496	-20.04	*-2.82	*-0.628	-	-0.18	*-0.15	*3.575	-0.237	*1.197
Phasianus colchicus	-0.043	*-0.616	*-1.303	0.011	*-0.367	*-0.329	*-1.323	*-2.704	*0.694	-	-0.021	*-0.152	-1.06	*-1.759	-0.235
Phylloscopus collybita	0.031	*-1.117	*-1.579	*-0.878	*-0.875	-0.16	*-1.597	*-1.386	0.149	-	*0.225	-0.021	*3.4	*1.327	-0.002
Phylloscopus trochilus	*0.497	*-0.768	*-0.924	*-0.854	*-0.802	0.071	*-1.325	*-2.408	*-1.074	-	0.144	-0.06	*3.431	0.81	0.366
Picus viridis	0.181	*-0.509	-0.177	*-0.569	*-0.661	0.02	-1.349	-17.05	*-0.778	-	-0.066	-0.004	-1.77	-0.133	-0.699
Prunella modularis	*0.422	*-0.653	*-1.024	*0.283	*0.396	*0.287	-0.258	0	*0.658	-	0.097	*0.073	-0.196	0.292	*0.31
Pyrrhula pyrrhula	0.341	*-1.676	*-2.272	*-0.627	*-0.329	0.022	-17.39	-1.087	0.343	-	-0.036	*-0.153	2.962	*2.615	0.198
Regulus regulus	*-0.534	*-2.096	*-2.529	*-1.707	*-0.796	*-0.898	*-3.227	*-2.328	*-0.538	-	-0.007	*-0.184	*3.5	0.305	*0.969
Sitta europaea	*-1.256	*-2.32	*-2.573	*-1.25	*-0.795	*-0.947	*-1.691	-21.74	*-1.427	-	-0.397	-0.112	2.748	0.011	0.181
Spinus spinus	*-0.408	*-2.898	*-1.983	*-1.706	*-1.272	*-1.712	-24.64	-25.02	*-2.21	-	-0.201	-0.099	1.833	0.035	*1.503
Streptopelia decaocto	*0.486	*-0.916	*-0.684	*0.346	*1.538	*0.727	0.475	-13.87	*1.225	-	0.043	*0.139	0.2	0.155	0.166
Sturnus vulgaris	*0.407	*0.762	*-0.94	*0.771	*1.493	*0.638	*0.62	-12	0.421	-	0.156	*0.499	1.546	1.115	0.081
Sylvia atricapilla	-0.068	*-1.163	*-2.074	*-0.81	*-0.832	*-0.215	*-1.921	*-1.546	0.267	-	0.114	-0.028	2.13	0.707	0.176
Sylvia borin	*0.524	*-1.307	*-1.457	*-0.999	*-1.176	0.121	-21.59	-21.5	-0.48	-	0.17	*-0.238	*7.115	1.054	0.306
Sylvia communis	*1.226	*0.707	0.283	*0.866	-0.044	*0.964	*0.572	*1.018	*5.772	*-4.652	0.166	*-0.073	1.037	0.067	*0.772
Sylvia curruca	*1.867	0.837	-0.407	*1.22	0.097	*1.402	-19.91	-19.3	*3.788	*-3.229	-0.1	-0.106	1.614	0.984	0.163
Tadorna tadorna	-0.350	0.847	-0.797	0.668	*1.5	*2.749	*1.514	-24.77	*1.967	-	-0.677	0.184	5.7	1.956	*3.796
Troglodytes troglodytes	*-0.221	*-1.082	*-1.154	*-0.653	*-0.555	*-0.12	*-1.277	*-0.554	-0.044	-	0.068	0.01	0.416	0.524	*0.265
Turdus merula	-0.023	*-1	*-1.471	*-0.29	*0.14	-0.091	*-0.593	*-0.655	*0.545	-	0.059	*0.077	0.834	0.46	0.153
philomelos	-0.043	*-1.21	*-1.808	*-0.77	*-0.469	*-0.236	*-1.732	*-1.599	-0.147	-	0.106	*-0.096	-1.531	-0.583	0.239
Turdus viscivorus	*-0.498	*-1.393	*-2.566	*-0.856	*-0.364	*-0.548	-17.72	-18.45	*-0.74	-	-0.091	-0.084	0.917	-0.465	-0.479
Vanellus vanellus	*1.024	*1.476	*0.731	*1.895	0.429	*1.913	-0.234	*2.278	*-1.04	-	0.266	-0.022	-3.273	*-3.331	-0.285

Supplementary Table 3. Results from separate analyses of major and minor roads. Effect sizes and *k* values for major and minor roads are given alongside those for the original associations with both road types together.

Scientific name	Common name	Roads together		Major roads		Minor roads	
		k	**Effect size**	***k***	**Effect size**	***k***	**Effect size**
Alauda arvensis	Eurasian skylark	4	-0.107	3	-0.057	4	-0.108
Anas platyrhynchos	Mallard	25	-0.288	60	-0.875	25	-0.306
Anthus pratensis	Meadow pipit	13	-0.243	11	-0.196	14	-0.264
Chloris chloris	European greenfinch	9	0.189	7	0.062	10	0.211
Columba livia domestica	Feral pigeon	7	0.123	70	-0.499	7	0.126
Corvus frugilegus	Rook	7	0.135	7	0.167	8	0.115
Corvus monedula	Eurasian jackdaw	9	0.128	40	0.605	8	0.103
Cyanistes caeruleus	Blue tit	2	0.006	25	-0.150	4	0.025
Emberiza citrinella	Yellowhammer	2	-0.010	25	-0.920	25	0.227
Fringilla coelebs	Common chaffinch	20	0.180	2	-0.015	20	0.191
Linaria cannabina	Common linnet	2	-0.017	3	-0.084	2	-0.015
Passer domesticus	House sparrow	9	0.281	14	-0.288	9	0.353
Phasianus colchicus	Ring-necked pheasant	2	-0.023	2	-0.047	2	-0.022
Phylloscopus collybita	Common chiffchaff	6	-0.025	4	-0.030	6	-0.021
Streptopelia decaocto	Eurasian collared dove	7	0.195	25	-0.297	7	0.235
Turdus merula	Common blackbird	5	0.042	35	-0.297	6	0.066

Supplementary Figures

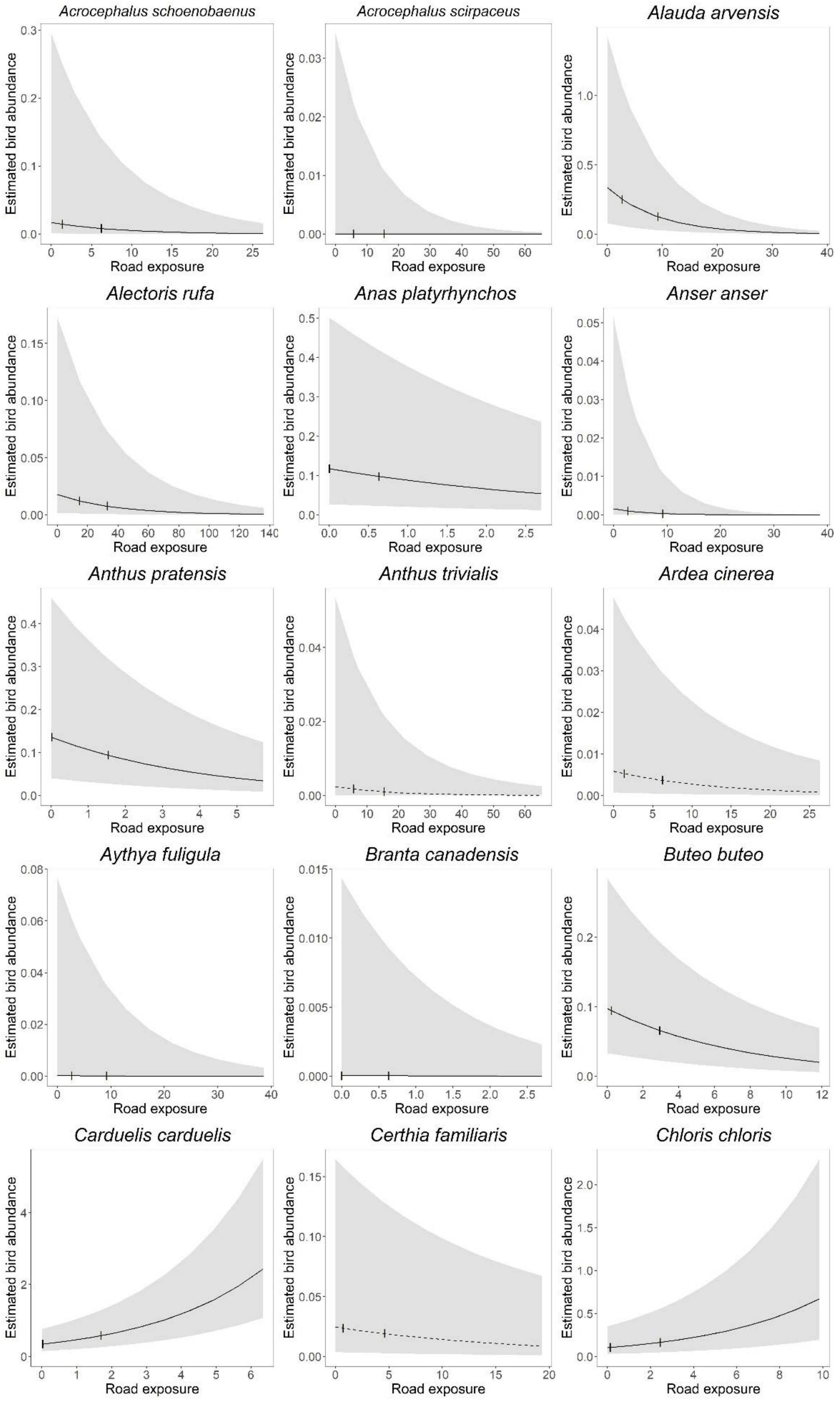

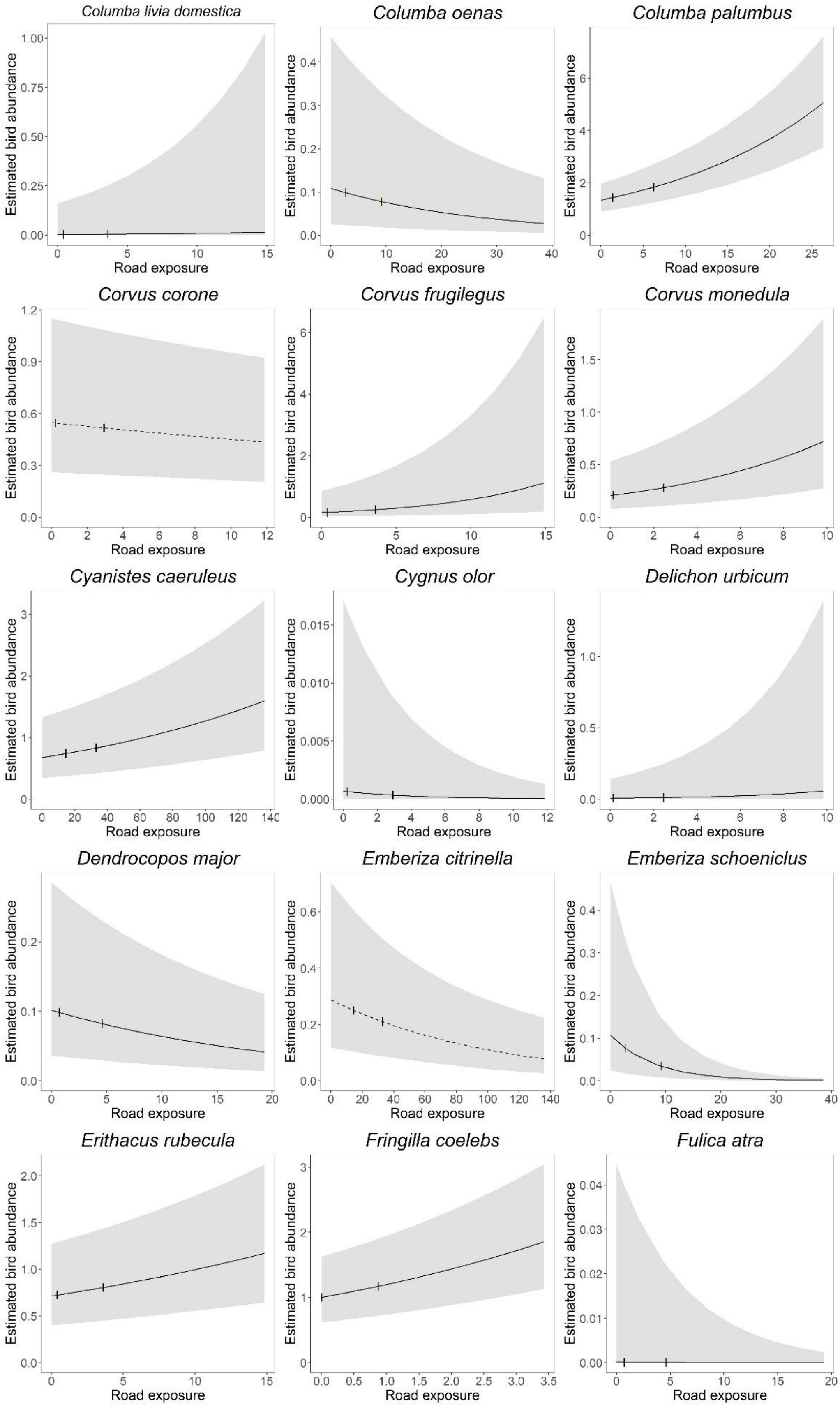
Columba livia domestica
Columba oenas
Columba palumbus
Corvus corone
Corvus frugilegus
Corvus monedula
Cyanistes caeruleus
Cygnus olor
Delichon urbicum
Dendrocopos major
Emberiza citrinella
Emberiza schoeniclus
Erithacus rubecula
Fringilla coelebs
Fulica atra
Estimated bird abundance
Road exposure

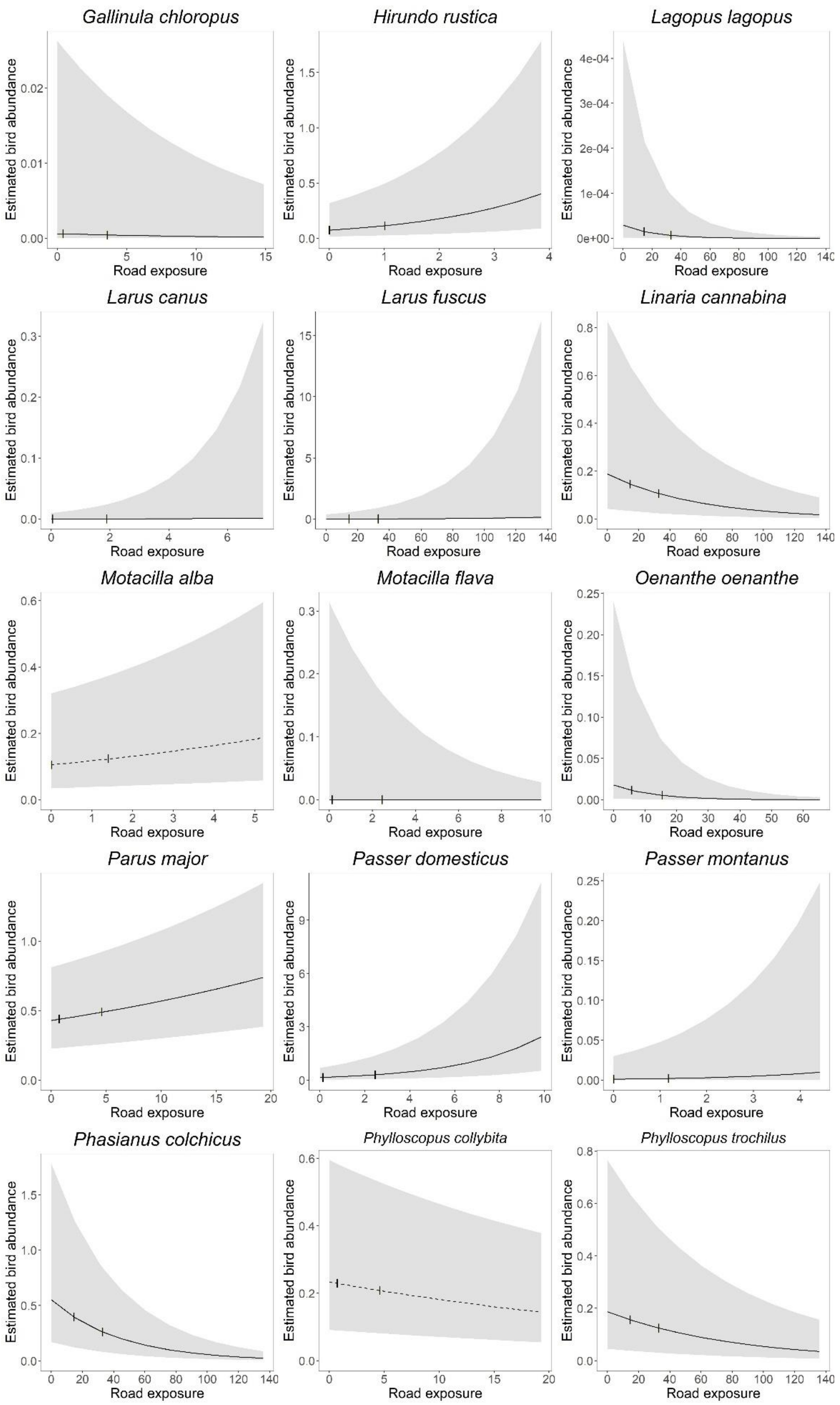
Gallinula chloropus
Hirundo rustica
Lagopus lagopus
Larus canus
Larus fuscus
Linaria cannabina
Motacilla alba
Motacilla flava
Oenanthe oenanthe
Parus major
Passer domesticus
Passer montanus
Phasianus colchicus
Phylloscopus collybita
Phylloscopus trochilus
Estimated bird abundance
Road exposure

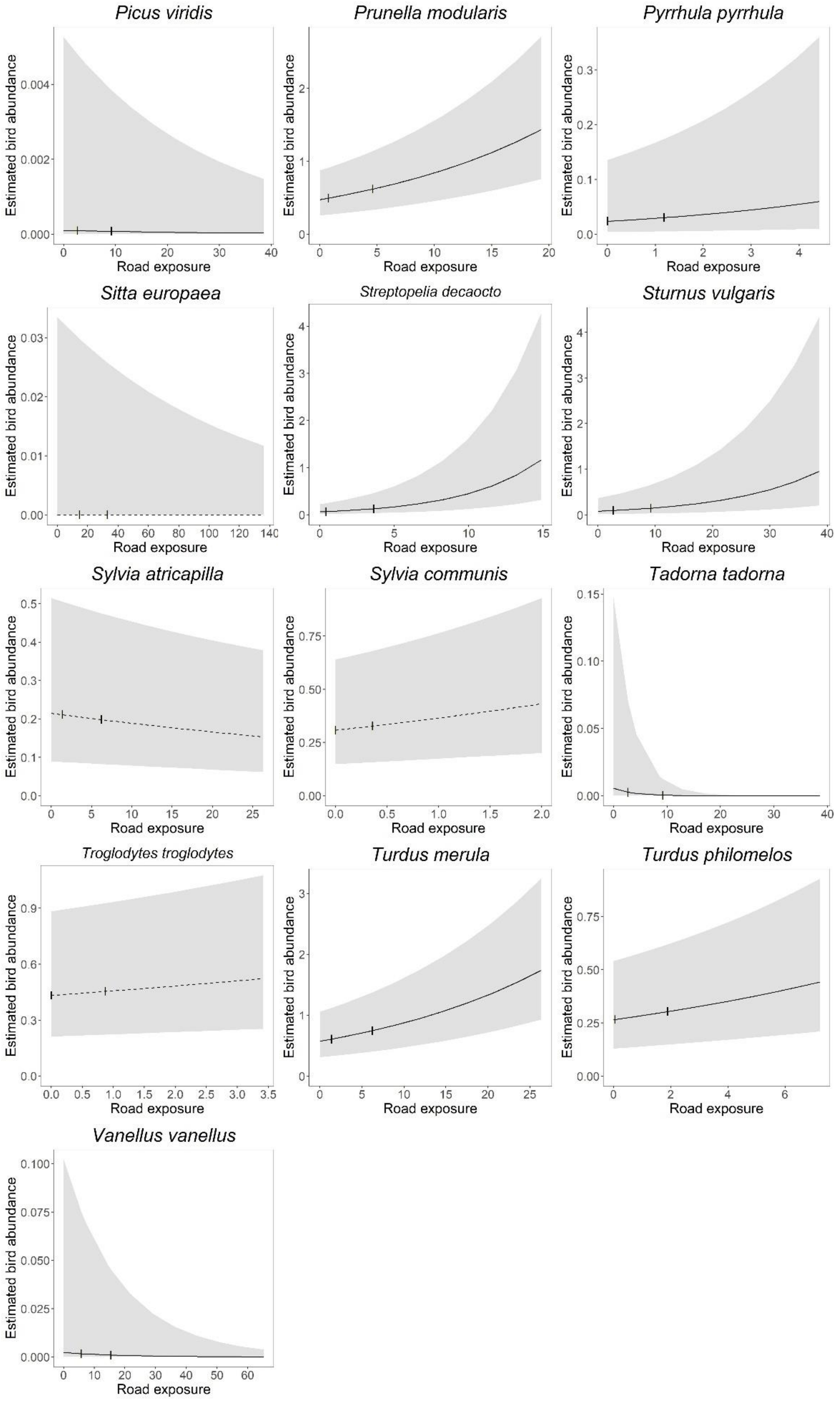

Supplementary Figure 1. Estimated bird abundance across the full ranges of road exposure. Estimated bird abundance was calculated for each species using its *k* value (which optimises the spatial scale between distance from road and road exposure). Y-axes represent estimated bird abundance within 100 m of a 200-m

BBS transect section. The 0.25 and 0.75 quartiles of road exposure for each species are indicated by the vertical lines. Only species with significant associations are shown here, and those that retained significance after Bonferroni correction are depicted by a solid, as opposed to dashed, line. Shaded areas denote 95% prediction intervals.

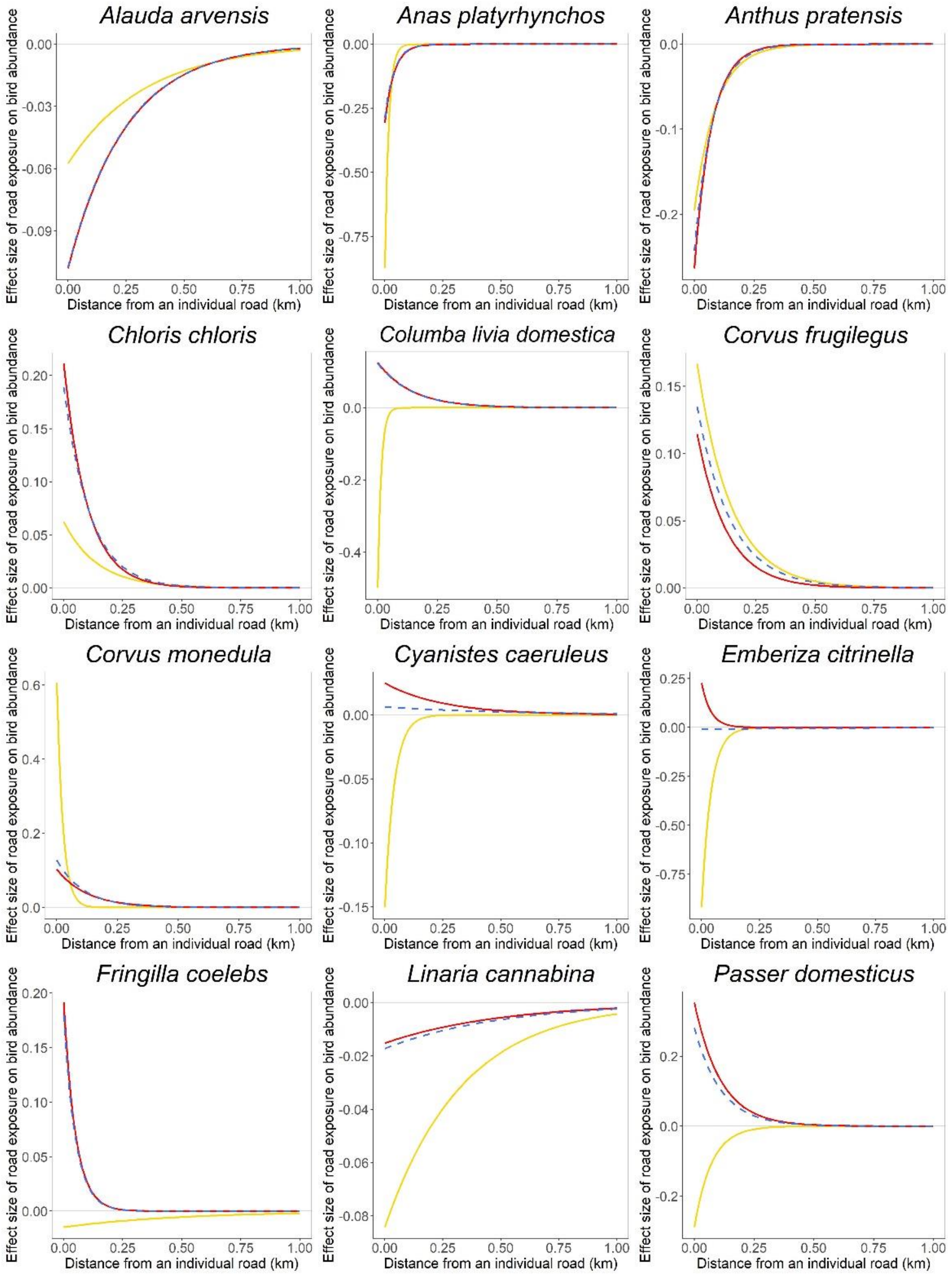

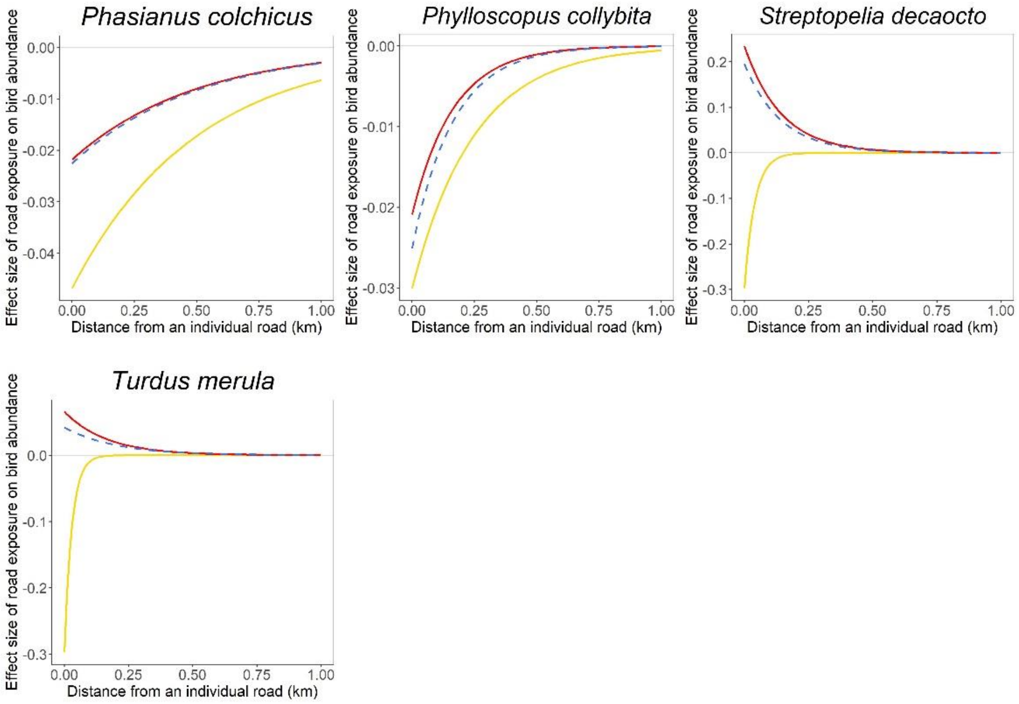

Supplementary Figure 2. Effect curves with distance from different road types. The intercept is determined by the coefficient and the rate of decline is determined by the parameter '*k*', which defines the spatial scale of the relationship between distance from road and road exposure for each species. Effect curves for major roads are shown in yellow, minor roads in red and both road types together in dashed blue.

Supplementary Methods

Calculating bird count, road exposure and other covariate values for the midpoint of every 200-m BBS transect

Bird count data

To produce bird count estimates for this study, we obtained data from the UK Breeding Bird Survey (BBS). For this survey 1-km squares are selected from those in the British National Grid using a stratified random sampling design[1]. Each square is surveyed annually in two early morning visits during the breeding season (early visit: beginning of April to mid-May; late visit: mid-May to end-June). On each visit, the surveyor walks two 1-km transects, each divided into five 200-m transect sections. These transects mostly do not run alongside roads (64% of the transect sections used in this analysis did not follow a paved road along any part of them). While walking a transect, surveyors note every bird they see or hear in each transect section, along with the estimated perpendicular distance of each bird from the transect (recorded as one of four distance bands: 0-25 m; 25-100 m; > 100 m; flying over the area). They also record the dominant habitat type in each 200-m transect section as one of nine broad classes: woodland (dominated by trees generally taller than 5 m); scrubland (dominated by woody shrubs or young trees shorter than 5 m); semi-natural grassland and marsh (dominated by grasses or by wet communities dominated by rushes/sedges/reeds etc); heathland and bogs; farmland (enclosed fields); human sites (areas associated with people i.e. buildings, parks and gardens); water bodies (freshwater); coastal; and inland rock. For full methods see BTO[1].

For our analyses, we extracted counts from BBS squares that had been surveyed every year from 2012-2014 inclusive. We selected three years to increase the sample size of counts for each species and to average-out the effect of annual population fluctuations whilst avoiding long-term abundance changes. Within each square, we excluded transect sections that did not have both habitat and route data recorded.

We then extracted counts of birds in the distance bands 0-25 m and 25-100 m, excluding those from the other two bands as they did not have both lower and upper distance limits. For each species, we then chose one focal survey visit to represent its breeding abundance – the early visit for resident species and the late visit for migrant species (**Supplementary Table 1**). We used counts from that visit to calculate the mean annual bird count in each transect section for that species, summing over both distance bands and averaging over years. Where transect sections had multiple habitats recorded across the years, we assigned a single habitat to each section, using the procedure outlined

in **Supplementary Figure 3**. Our final dataset contained counts from 19,709 200-m transect sections, in 2,033 1-km BBS squares spread widely across Britain (**Supplementary Figure 4**).

We then excluded species with a total mean annual bird count < 100, as preliminary analysis showed this to be the minimum sample size required to successfully run our models. We also excluded two winter flocking species: fieldfare *Turdus pilaris* and golden plover *Pluvialis apricaria*, as their flocks can persist into the start of the breeding season, potentially affecting model results. Finally, we removed any transects with mean annual counts > 10 for three wading bird species: Eurasian curlew *Numenius arquata*, northern lapwing *Vanellus vanellus* and Eurasian oystercatcher *Haematopus ostralegus*, to exclude counts from non-breeding flocks. This process gave us mean annual count estimates for 79 species at every 200-m transect section. As we chose to use counts within 100 m of each transect section (from the 0-25 m and 25-100 m distance bands), each mean annual count estimate pertained to a maximum area of 200 m x 200 m.

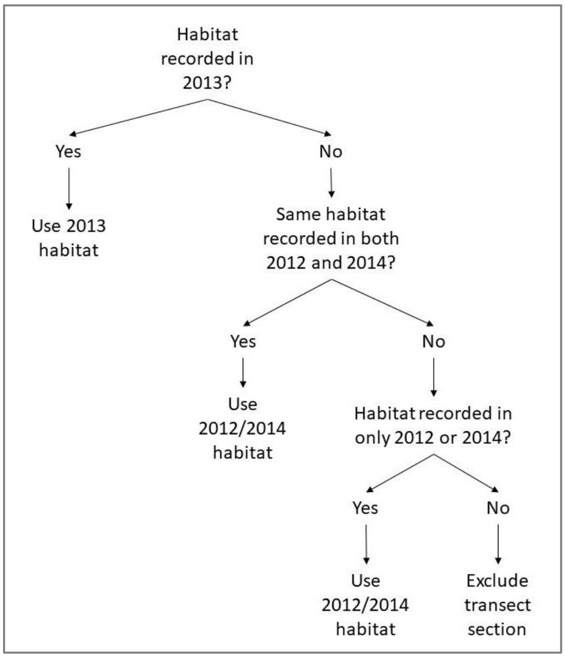

Supplementary Figure 3. Habitat assigning methodology. This procedure was used to assign a single habitat class to transect sections with multiple habitats recorded across the three-year period. This figure was first published in Cooke et al. 2020[2] and is reproduced here under a CC BY 4.0 licence[3].

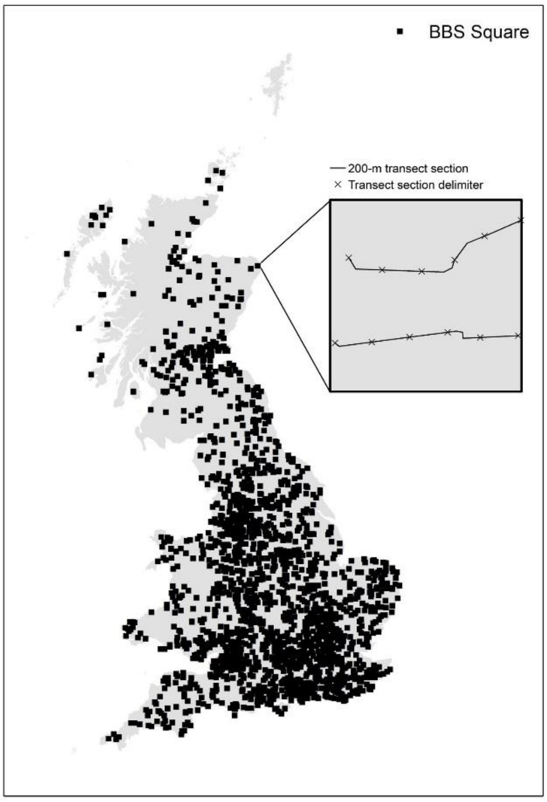

Supplementary Figure 4. The locations of BBS squares used in this study. The inset gives an example of the layout of a BBS square, crossed by two 1-km transects. Great Britain boundary shapefile obtained from ONS[4]. This figure was first published in Cooke et al. 2019[5] and is reproduced here under a CC BY 4.0 licence[3].

Road data

We obtained shapefiles for all paved road classes in every region of England, Scotland and Wales, excluding the Isles of Scilly, from the Ordinance Survey Open Data's[6] Meridian 2 dataset for January 2013 (Meridian 2 v1.2 Release 1 2013). This was supplied by Global Mapping Ltd[7].

To estimate the road exposure at each BBS transect section midpoint, we used the following methods. First, we placed points (hereafter kernel points) at intervals of 100 m along every road. Then, we calculated the distances between each BBS 200-m transect section midpoint and all kernel points within a 5-km radius of that midpoint (**Supplementary Figure 5**). We chose the distance of 5-km on the assumption it was greater than the maximum distance that any effect of a road on bird abundance would reach.

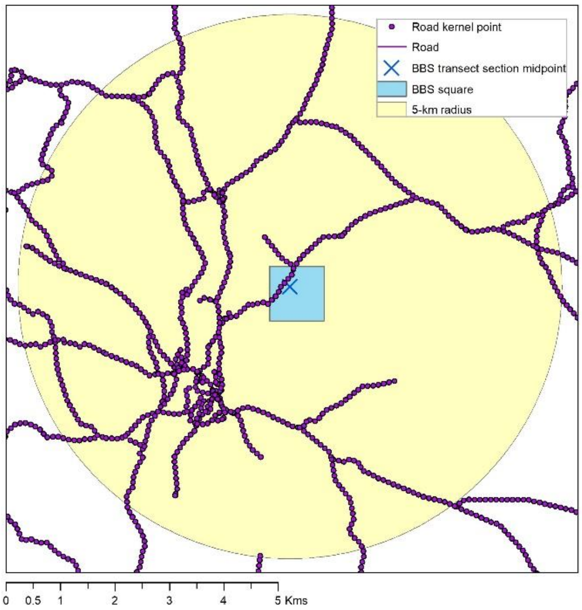

Supplementary Figure 5. Radii for calculating road exposure. Each 5-km radius was placed around the midpoint of a 200-m BBS transect section within a BBS square. All roads within each radius had kernel points placed along them, at every 100 m.

As some road impacts are likely to act on birds in areas around roads (e.g. noise disturbance and habitat effects), but others only on or over the road surface itself (e.g. collisions and perching opportunities), we assumed a negative exponential relationship between distance from a road and the exposure of a transect midpoint to that road, with road exposure being highest on the road itself and declining with distance. We used the following formula to calculate road exposure at each transect midpoint:

Exposure to roads = $\sum(\exp(d_i * -k))$

Where:

d_i *= distance from the midpoint of the transect section to kernel point i*

k = parameter determining the scale of the relationship between road exposure and distance from a road

We chose not to incorporate traffic volume data in these calculations as high-resolution traffic data is only collected for motorways and A-roads in Britain. In the above equation, as *k* increases, road exposure at distance *d* decreases (**Supplementary Figure 6**). The values of *k* for each species were optimised within the GAMM framework by running multiple GAMMs with a range of *k* values and selecting the value which produced the model with the highest log-likelihood. The values of *k* we tested ran from 1-100. We chose these limits as these assume that road exposure approaches zero at approximate distances of 5 km and 0.05 km respectively, which we thought were above and below the distance expected in reality. We did not evenly space the *k* values, due to the non-linear

relationship between *k* and distance, but tested values of: 1-20 in increments of 1; 25-45 in increments of 5; and 50-100 in increments of 10. If no peak in log-likelihood could be identified, we did not continue with the analysis for that species. This resulted in three species being excluded: corn bunting *Emberiza calandra*, common redstart *Phoenicurus phoenicurus* and Eurasian magpie *Pica pica*. We later removed one further species, ring-necked parakeet *Psittacula krameria*, as a reliable model for this species could not be produced, leaving 75 species in the analysis.

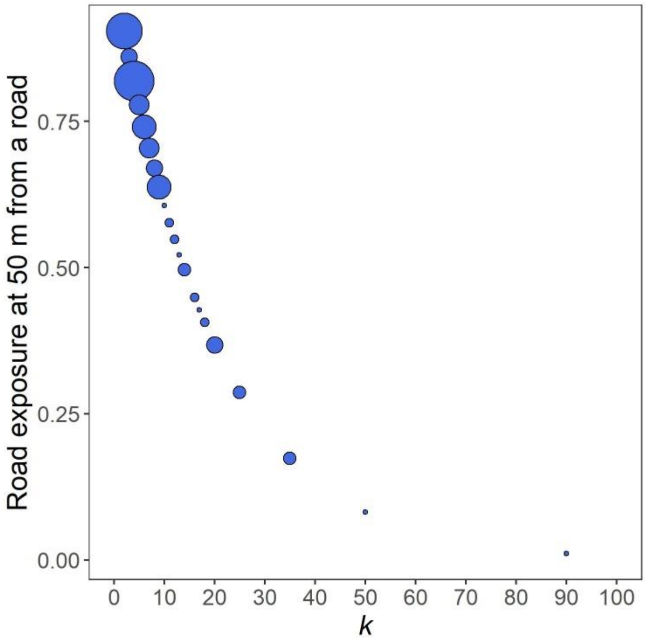

Supplementary Figure 6. Relationship between *k* and road exposure. Road exposure is calculated at 50 m from a single road, for the optimum values of *k* identified for each of the 75 species. Point size represents the frequency of the *k* value.

Arable land

Arable yield, which effectively captures a range of metrics describing agricultural intensity, can have a large effect on bird density[8]. High-resolution yield data were not available for the whole of our study area, so we derived a proxy using CEH's 2015 Land Cover Map vector dataset[9] from the Edina Environment Digimap Service[10]. We extracted the 'arable land' habitat class and calculated the proportion of arable land within 5-km buffers centred on the midpoint of each BBS 200-m transect section. To confirm that this was a suitable proxy for arable yield, we calculated the same measure for 2,254 1-km squares in the east and south of England, for which we were able to obtain yield estimates from Finch et al.[11]. These were derived independently using a combination of Farm Business Survey data[12] and farm owner surveys. We found a strong positive correlation between the two (Pearson's correlation coefficient = 0.82). We also calculated the area of the largest arable polygon (corresponding to cropping unit or field) in each 5-km buffer as an additional measure of

agricultural heterogeneity across our study area (Pearson's correlation coefficient with yield estimates = 0.34).

Other covariates

We obtained gridded local resident human population density estimates at a 1-km^2 spatial resolution, from the Centre for Ecology and Hydrology[13]. We $\log_{10}$-transformed these data as we assumed the difference in potential impact from e.g. 1-1000 people would be greater than that from 10,000-11,000 people. To account for spatial variation in weather, we obtained temperature and rainfall data from the Met Office[14] for ten regions across Great Britain. We used these to calculate the mean temperature and rainfall during the survey season (April-June), in our three-year period. We obtained tree cover density estimates for 2012 (in the form of cover percentage from 0-100% at a spatial resolution of 100 m, estimated using high-resolution satellite data) from the Copernicus Land Monitoring Service[15]. To the midpoint of each 200-m BBS transect section, we then attributed the $\log_{10}$-transformed human population density of the 1-km grid square it lay in, the temperature and rainfall values for the region it was in, and the mean of the 100 m^2 tree cover density estimates within a surrounding 5-km buffer. Our reasoning for incorporating tree cover density in this way was to capture habitat effects on a more landscape scale than the local habitats recorded in the BBS surveys and to match the distance over which the proportion of arable land and the road exposure variables were measured.

Comparing results of common species with and without detectability incorporated

Cooke et al.[5] demonstrated the importance of accounting for detectability in producing accurate estimates of the associations between road exposure and bird populations. As sample size limitations prevented us from doing so for all the species in our analysis, we reproduced the GAMMs for 50 common species with detectability incorporated, to ascertain the difference.

We fitted distance sampling models (using the R package "mrds"[16]) to the count data for each species, using raw count at each 200-m transect section as the response and both habitat and road exposure as covariates. As the bird count data were from only two distance bands, we used a half-normal detection function with no adjustment. Within this set up, we optimised the spatial component of the road exposure variable, *k*, in the same way as in the main analysis – running iterations of the model with values of *k* from 1-100 and choosing the value that produced the peak log-likelihood. We removed two species from this sub-analysis here as no optimum value of *k* could be identified. Using these distance sampling models, we estimated detectability at each 200-m BBS transect section. We then reproduced the GAMMs, analysing the associations between abundance and road exposure, for the remaining 48 species, but this time incorporating the estimated

detectability as an offset. This resulted in only small modifications to the effect size estimates for all species and a change of effect direction for only two species, coal tit *Periparus ater*, and carrion crow *Corvus corone*, both of which did not have significant (after Bonferroni correction) associations between road exposure and abundance with or without detectability included (**Supplementary Figure 7**).

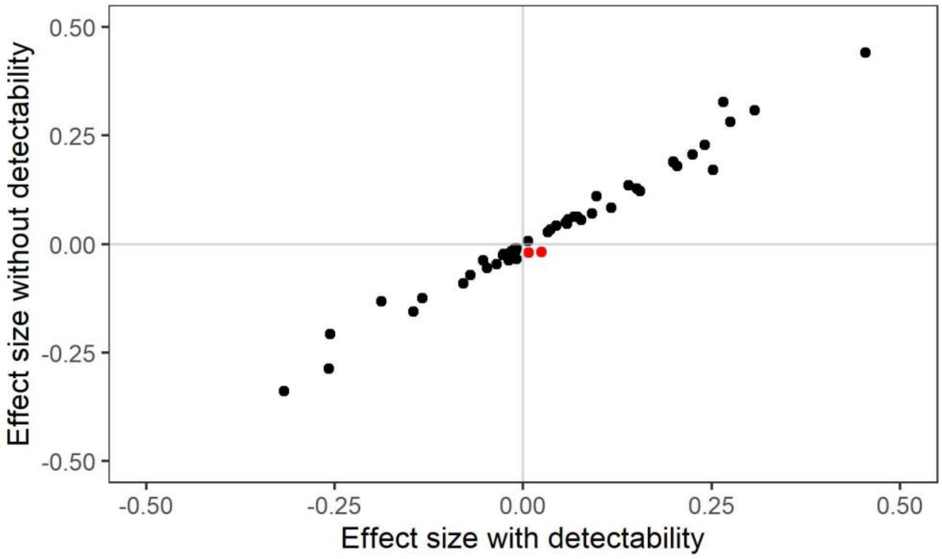

Supplementary Figure 7. The effect of detectability inclusion. The effect sizes of road exposure on bird abundance were compared with and without detectability included as an offset. Two species that showed changes in effect direction, coal tit and carrion crow, are shown in red.

Supplementary References

1. BTO. Methodology and survey design. https://www.bto.org/volunteer-surveys/bbs/research-conservation/methodology (Accessed 2019).
2. Cooke, S.C., Balmford, A., Johnston, A., Newson, S.E. & Donald, P.F. Variation in abundances of common bird species associated with roads. *J. Appl. Ecol.* **00**, 1-12 (2020).
3. Creative Commons. https://creativecommons.org/licenses/by/4.0/ (2020).
4. ONS. Open Geography Portal. Countries (December 2014) Boundaries UK BFE. Office for National Statistics licensed under the Open Government Licence v.3.0. http://geoportal.statistics.gov.uk. Contains OS data © Crown copyright and database right (2014).
5. Cooke, S.C., Balmford, A., Johnston, A., Massimino, D., Newson, S.E. & Donald, P.F. Road exposure and the detectability of birds in field surveys. *Ibis* (2019).
6. OS. Open Data. Ordnance Survey licensed under the Open Government Licence v.3.0. https://www.ordnancesurvey.co.uk/business-and-government/products/opendata.html. Contains OS data © Crown copyright and database right (2013).
7. Global Mapping Ltd. OS Meridian 2. http://www.globalmapping.uk.com/gm_updates0505/meridian2. (Accessed 2016).
8. Donald, P. F., Sanderson, F. J., Burfield, I. J. & van Bommel, F. P. J. Further evidence of continent-wide impacts of agricultural intensification on European farmland birds, 1990–2000. *Agr. Ecosyst. Environ.* **116**, 189–196 (2006).
9. Rowland, C. S. *et al.* Land Cover Map 2015 (vector, GB). NERC Environmental Information Data Centre. https://catalogue.ceh.ac.uk/documents/6c6c9203-7333-4d96-88ab-78925e7a4e73 (2017).
10. Edina. https://digimap.edina.ac.uk/environment (Accessed 2018).
11. Finch, T. *et al.* Bird conservation and the land sharing-sparing continuum in farmland-dominated landscapes of lowland England. *Conserv. Biol.* **33**, 1045–1055 (2019).
12. Farm Business Survey (FBS). http://www.farmbusinesssurvey.co.uk. (2019).
13. Reis, S. *et al.* UK gridded population 2011 based on Census 2011 and Land Cover Map 2015. NERC Environmental Information Data Centre. (Dataset). https://doi.org/10.5285/0995e94d-6d42-40c1-8ed4-5090d82471e1 (2017).
14. Met Office. Meteorological Office licensed under the Open Government Licence v.3.0. https://www.metoffice.gov.uk/climate/uk/summaries/datasets (2018).
15. Copernicus. https://land.copernicus.eu/pan-european/high-resolution-layers/forests/tree-cover-density/status-maps/2012. © European Union, Copernicus Land Monitoring Service 2012, European Environment Agency (EEA) (Accessed 2018).
16. Laake, J., Borchers, D., Thomas, L., Miller, D. & Bishop, J. *mrds: Mark-Recapture Distance Sampling. R package version 2.1.18.* (2017).
