# Supplementary material for: Roads as a contributor to landscape-scale variation in bird communities
Source: Nat Commun. 2020 Jul 7;11:3125. doi: 10.1038/s41467-020-16899-x (PMC7340783; doi:10.1038/s41467-020-16899-x)
Supplement: Supplementary file 3 — Reporting Summary [file 41467_2020_16899_MOESM3_ESM.pdf]

nature research

Corresponding author(s): Sophia C. Cooke

Last updated by author(s): Mar 4, 2020

Reporting Summary

Nature Research wishes to improve the reproducibility of the work that we publish. This form provides structure for consistency and transparency in reporting. For further information on Nature Research policies, see Authors & Referees and the Editorial Policy Checklist .

Statistics

For all statistical analyses, confirm that the following items are present in the figure legend, table legend, main text, or Methods section.

n/a	Confirmed	
☐	☒	The exact sample size (*n*) for each experimental group/condition, given as a discrete number and unit of measurement
☐	☒	A statement on whether measurements were taken from distinct samples or whether the same sample was measured repeatedly
☐	☒	The statistical test(s) used AND whether they are one- or two-sided<br>*Only common tests should be described solely by name; describe more complex techniques in the Methods section.*
☐	☒	A description of all covariates tested
☐	☒	A description of any assumptions or corrections, such as tests of normality and adjustment for multiple comparisons
☐	☒	A full description of the statistical parameters including central tendency (e.g. means) or other basic estimates (e.g. regression coefficient) AND variation (e.g. standard deviation) or associated estimates of uncertainty (e.g. confidence intervals)
☐	☒	For null hypothesis testing, the test statistic (e.g. *F*, *t*, *r*) with confidence intervals, effect sizes, degrees of freedom and *P* value noted<br>*Give P values as exact values whenever suitable.*
☒	☐	For Bayesian analysis, information on the choice of priors and Markov chain Monte Carlo settings
☒	☐	For hierarchical and complex designs, identification of the appropriate level for tests and full reporting of outcomes
☐	☒	Estimates of effect sizes (e.g. Cohen's *d*, Pearson's *r*), indicating how they were calculated

Our web collection on statistics for biologists contains articles on many of the points above.

Software and code

Policy information about availability of computer code

Data collection	We wrote custom R scripts to collate and prepare the data for this study and will make these available on Apollo, the University of Cambridge's data repository.
Data analysis	We wrote custom R scripts to analyse the data for this study and will make these available on Apollo, the University of Cambridge's data repository.

For manuscripts utilizing custom algorithms or software that are central to the research but not yet described in published literature, software must be made available to editors/reviewers. We strongly encourage code deposition in a community repository (e.g. GitHub). See the Nature Research guidelines for submitting code & software for further information.

Data

Policy information about availability of data

All manuscripts must include a data availability statement. This statement should provide the following information, where applicable:

- Accession codes, unique identifiers, or web links for publicly available datasets
- A list of figures that have associated raw data
- A description of any restrictions on data availability

The data that support the findings of this study will either be provided on the Apollo repository, or they are freely available online, in which case links to their original sources will also be provided on Apollo.

Field-specific reporting

Please select the one below that is the best fit for your research. If you are not sure, read the appropriate sections before making your selection.

☐ Life sciences ☐ Behavioural & social sciences ☒ Ecological, evolutionary & environmental sciences

For a reference copy of the document with all sections, see nature.com/documents/nr-reporting-summary-flat.pdf

Ecological, evolutionary & environmental sciences study design

All studies must disclose on these points even when the disclosure is negative.

Study description	We analysed associations between bird abundance and road exposure across Great Britain, in relation to species characteristics.
Research sample	We used bird count data from the UK Breeding Bird Survey (BBS). We did not collect any count data ourselves.
Sampling strategy	As we used the UK BBS, our study covers the whole of Britain. We only analysed species that, in preliminary analyses, had sample sizes large enough for successful model convergence.
Data collection	The UK BBS is a volunteer survey run by the British Trust for Ornithology, the Royal Society for the Protection of Birds and the Joint Nature Conservation Committee. Only experienced volunteers participate in this survey.
Timing and spatial scale	We used data from the BBS which spanned three years, 2012-2014.
Data exclusions	We excluded winter-flocking species, as their flocks can persist into Spring, altering model results.
Reproducibility	We have made all our code accessible and all the data we used is available either online, or from the British Trust of Ornithology.
Randomization	The study did not require randomization as we used data already collected and analysed data for each species separately.
Blinding	The study did not require blinding as we used data already collected.

Did the study involve field work? ☐ Yes ☒ No

Reporting for specific materials, systems and methods

We require information from authors about some types of materials, experimental systems and methods used in many studies. Here, indicate whether each material, system or method listed is relevant to your study. If you are not sure if a list item applies to your research, read the appropriate section before selecting a response.

Materials & experimental systems

n/a	Involved in the study	
☒	☐	Antibodies
☒	☐	Eukaryotic cell lines
☒	☐	Palaeontology
☒	☐	Animals and other organisms
☒	☐	Human research participants
☒	☐	Clinical data

Methods

n/a	Involved in the study	
☒	☐	ChIP-seq
☒	☐	Flow cytometry
☒	☐	MRI-based neuroimaging
