# Supplementary material for: Roads as a contributor to landscape-scale variation in bird communities
Source: Nat Commun. 2020 Jul 7;11:3125. doi: 10.1038/s41467-020-16899-x (PMC7340783; doi:10.1038/s41467-020-16899-x)
Supplement: Supplementary file 2 — Peer Review [file 41467_2020_16899_MOESM2_ESM.pdf]

Reviewers' comments:

Reviewer #1 (Remarks to the Author):

The extent to which road networks influence and impact biodiversity is usually considered at specific locations of interest, limited to a few key species and based on short duration data. The actual geographical scale of roads, especially in places such as the UK would appear to be simply overwhelming in their extent and threat. This study takes all of this seriously and, by combining geographical data with long-term and detailed data on bird populations, provides an extraordinary - and alarming - image of the way in which transportation infrastructure - an anthropogenic construct on a global scale yet one effectively invisible through its ubiquity - has impacted on birds at a national level.
This study is sound, significant and devastating. It is a story that needs to be told.

Reviewer #2 (Remarks to the Author):

Comments on manuscript 'Roads as a contributor to landscape-scale variation in bird communities', Cooke et al.

General comments
This paper is addressing a very interesting and relevant topic, the relation between road density and the composition of bird communities. It is a very thorough study with a nice and large dataset over a large area. The design and statistical analyses have been carried out excellently with lots of checks and balances. The paper is very concise and well-written. I think the results are robust and give a good impression of how road density explains variation in bird communities and the conclusions are justified. The paper is novel in the sense that it addresses the impacts of roads over a large scale with a representative selection of birds in the breeding season. It looks at the total road density instead of only focussing on highways or on the contrary small country roads. However, this complicates also the interpretation of the results. Small country roads have another impact than large highways and can even cause opposite effects for the same species, e.g. a goose species might profit from breeding in the high noise load zones but relative low (human presence) disturbance zones directly along a highway but might avoid zones along a small country road with more recreational use. The authors clearly are aware of this and added an analysis on a subset of minor roads. My only major comment and question is why they did not include an analysis on a subset with roads/highways with high traffic intensity, even if the data allow only analyses for a smaller set of species. The hypothesis would be that the results show more negative impacts and larger disturbance distances in that analysis and if confirmed this would clearly add to the robustness of the study and facilitate interpretation of the results.
A minor general remark is that I miss some insight in what exactly the models are that were tested and what the results are. For instance I would like to see a table showing the results of the basic GAMM models applied and not only the results for the effect of road exposure.

Specific comments
Line 44: I suggest to add visual disturbance
Line 46: I think the evidence points out that a few kilometres is exceptional but distances up to many hundreds of meters are often mentioned, maybe better to state what is the most commonly found magnitude rather than stressing the extremes
First 2 paragraphs: maybe good to add a few remarks to the fact that road encroachment often goes together with habitat changes and also intensity of use by human activities. Often difficult to tear apart from the pure road (use) effects.
Line 47-48: I think it is also important to mention that you study áll paved roads and not only heavily travelled roads. It is more about the density of roads than the effect of a single (heavily used) road.
Line 70: as start of the results section I would like to see a reference to an overview of the basic

results of the GAMS (in a table in supplementary section). So what are the significant variables per species?
Line 110. I am interested in the statistical effect of the land use intensity parameters. Were they indeed significant and do you think you corrected sufficiently for it?
Line 124, table 1: it seems to me that mean body mass and national population size are confounded, even if the correlation is not so high it hampers inclusion of both how do the authors interpret this? And did they explore the effect of an interaction term?
Line 145: noise effects: also Halfwerk et al. (2011), disturbance effects: I think the main impacts particularly along smaller roads in the country side with recreational use (walkers and bikers) is caused by visual disturbance. There is a wide range of evidence indicating that certainly larger bodied species (e.g. waterfowl and waders) have 'flight initiation distances' up to many hundreds of meters (Livezey et al. 2016)
Line 147: likewise for open land ground nesting birds (like meadowbirds) these structures associated with roads are causing also disturbance effects (van der Vliet et al. 2010).
Line 158: Collisons seems an unlikely explanation for disturbance effects up to 500 meter. The evidence points to a high impact of noise disturbance along higways in the case of songbirds. And along smaller roads the lower intensity of cars also will lead to a much lower risk of collisions.
Line 164: what is the likelihood that positive effects found in your analyses can be attributed to small scale subtle differences in habitats along roads or availability food/grit? Instead of only focussing on a quite formal statistical analysis I would like to have a bit more of ecological understanding/discussion of why species react like they do. This would add to the discussion/conclusion whether you think it is indeed the road/traffic that is causing effects or whether roads act as a proxy for (subtle) land use changes.
Line 165-167: Also with regards to the previous remarks I think this conclusion is not justified. Specialisation index is not automatically correcting for habitat differences linked to roads within the broad BBS habitat categories so there still is a possibility that roads are proxy for subtle habitat differences. Furthermore human disturbance is a container for all kinds of types of disturbance (noise, visual disturbance, light) so it is not clear to me how this tolerance would work. I think many of the shown associations remain a black box and therefore explanations for the observed relations remain very speculative
Line 169: there is some evidence for a lowered habitat quality (e.g. studies Reijnen cs).
Line 185: is this 600 meter only for the negative impacts? Since the authors talk about conservation, declines and extinction that would justify quantifying this for only the negative impact species. Even better, also showing this for roads with heavy traffic for which maybe larger disturbance zones might be expected.
Line 188. See also Reijnen et al. (1997) for recommendations of meadow bird conservation in relations to proximity of roads.
Line 220: Would it be possible to add a figure showing the relation between k and an 'impact distance' so the reader can interpret the k-values better?
Line 228: but where can I see the results of the effects of these covariates?

Supplementary section S1
Table 1.1. What is survey visit E and L? I suspect Early and Late, so please add to table heading
Line 238: I do not see this in table S1.1

Supplementary section S2
I wonder how many BBS squares are located in urban areas and how the obvious higher density of roads in urban habitat is influencing the results. Have the authors tested this (e.g. by omitting urban habitats from the analyses)?
Also I am curious how many (%) transect sections are at close range of a road and even more interestingly, how many are in the close proximity of a heavily travelled road. Can you be confident that the analyses have sufficient power to detect effects. Could you show some simple statistics about that?
About the habitat descriptors: what is meant by human sites, urban areas, industrial sites, railways???

Reijnen, R, Foppen, R & Veenbaas, G (1997) Disturbance by traffic of breeding birds: evaluation of the effect and considerations in planning and managing road corridors. Biol Cons 6, 567-581.
van der Vliet R.E., van Dijk J. & Wassen M.J. (2010) How different landscape elements limit the breeding habitat of meadow bird species. ARDEA 98 (2) : 203 - 209.
Livezey, Kent B., Esteban Fernández-Juricic, and Daniel T. Blumstein. 2016. "Database of Bird Flight Initiation Distances to Assist in Estimating Effects from Human Disturbance and Delineating Buffer Areas." Journal of Fish and Wildlife Management 7(1): 181–91.
W Halfwerk, LJM Holleman, CKM Lessells, H Slabbekoorn (2011). Negative impact of traffic noise on avian reproductive success. Journal of applied Ecology 48 (1), 210-219

Reviewer #3 (Remarks to the Author):

Summary

This paper explored the effects of road density on bird populations across Great Britain. Anthropogenic influences on species habitat is a global ecological concern, and while the insights of this study are fairly local in nature, it is easy to grasp the relevance of the issues and understand how the methodology could be repeated elsewhere and for other taxonomic groups.

Simply put, I thought this was a fantastically written manuscript. The authors clearly put great care into organizing their ideas, motivating the research, explaining their methodology, and communicating their findings. The study is presented in a very accessible manner, and I think a broad group of readers will find it interesting. If all manuscripts were this well polished for submission, I would take great joy in being a more frequent reviewer.

I find no major problems with the authors' methods or findings, and can wholeheartedly recommend publication of this manuscript with some minor revisions.

General comments:

I think the manuscript would benefit from a paragraph that zooms in on an individual species or two (perhaps ones with opposite responses for narrative contrast). It would be neat to see individual species observations mapped out in relation to the road network to illustrate the types of relationships the authors describe.

Likewise, perhaps a bit of additional discussion about the generalizability of these results to the rest of the globe. Is Great Britain representative of broader patterns of avian diversity? Should the reader expect the same relationships to be found elsewhere? Where are the locations that are under greatest threat from road development and human growth, and how do those locations correspond with avian abundance?

Line comments:

Lines 31 – 34: This sentence is a bit clunky as written. Additionally, the term "biodiversity" is quite broad, and can mean different things in different contexts. Perhaps reword as something like "It has been argued that differential responses in species' abilities to cope with anthropogenic disturbance are leading to reductions in beta diversity…" or "It has been argued that differential responses in species' abilities to cope with anthropogenic disturbance are leading to a homogenization of species assemblages…"

Furthermore, the introductory paragraph introduces the concept of biotic homogenization, but as far as I can tell, the study does not attempt to measure or quantify homogenization in any way. In fact, this is also reflected in the manuscript title by the use of the word "communities". While the discussion comes back around and does a good job of contextualizing the results within this concept, I can't help but feel left a little wanting. It would be neat to see something that compares road density with species assemblages, for example.

Lines 38 – 39: It might be easy for the casual reader to incorrectly compare the figures in this sentence and conclude small increases (e.g., 1.3 billion to 1.5 billion). I suggest changing the latter figures to state the total expected number of road kms and vehicles rather than the increase (i.e., 95 million km of roads and 2.8 billion vehicles by 2050).

Line 56: There are presumably many ways in which to derive a habitat specialization index, so I think it would make more sense to say "an index of habitat specialization" rather than simply "habitat specialization index".

Lines 56 – 57: The study appears to have been conducted for the entirety of the island of Great Britain, but one of the predictors is described as "UK national population size". Do these population sizes include bird populations of Northern Ireland? If not, consider renaming the predictor for clarity. (Caveat: I'm a woefully ignorant Yank when it comes to these kinds of political subtleties; if what I just said makes no sense, please feel free to disregard.)

Line 61: Reword slightly to "…previous work has shown migrant populations to be reduced…"

Line 71: Reorder to "We calculated the road exposure… …within a 5-km radius of each site using kernel density estimation."

Line 72: When I read the words "composite", "density", and "paved roads" all in one sentence, my mind bizarrely tried to interpret the statement as having something to do with the physical structure of the road itself. Perhaps change to "…a combination of the geographic density and distance of all paved roads…".

Lines 76 – 77: What about species with fewer than 500 individual birds? Does this bias the study toward more common birds? How does the UK BBS identify individual birds over a three-year period and avoid double-counting?

Line 100, Figure 2: I think a little more could be done with this figure, as it currently only really conveys a single dimension of data. For example, instead of the x symbols, you might use circles that are colored with a gradient determined by the effect sizes from Figure 1; there is some interesting reordering of species, suggesting that the largest effect sizes do not necessarily translate to the largest changes in abundance. And/or use point size to indicate total population size. It would probably be beneficial to condense the vertical space in this plot, as there is a considerable amount of white space. Also, consider removing the horizontal bars, as they don't actually contribute any new information.

Lines 206 – 213: From the methods description, it does not appear that there is any distinction made between individual birds, and that the >500 number refers to summed population counts over the three-year study period. Given that individual birds are almost surely being counted multiple times across surveys, I would suggest rewriting this to avoid the use of "individual birds". The same suggestion applies to Lines 76 – 77.

Signed,
D. Scott Rinnan

Reviewer #1 (Remarks to the Author):

The extent to which road networks influence and impact biodiversity is usually considered at specific locations of interest, limited to a few key species and based on short duration data. The actual geographical scale of roads, especially in places such as the UK would appear to be simply overwhelming in their extent and threat. This study takes all of this seriously and, by combining geographical data with long-term and detailed data on bird populations, provides an extraordinary - and alarming - image of the way in which transportation infrastructure - an anthropogenic construct on a global scale yet one effectively invisible through its ubiquity - has impacted on birds at a national level.

This study is sound, significant and devastating. It is a story that needs to be told.

We thank the reviewer for this positive and kind feedback on our work – it was lovely to read.

Reviewer #2 (Remarks to the Author):

General comments

This paper is addressing a very interesting and relevant topic, the relation between road density and the composition of bird communities. It is a very thorough study with a nice and large dataset over a large area. The design and statistical analyses have been carried out excellently with lots of checks and balances. The paper is very concise and well-written. I think the results are robust and give a good impression of how road density explains variation in bird communities and the conclusions are justified. The paper is novel in the sense that it addresses the impacts of roads over a large scale with a representative selection of birds in the breeding season. It looks at the total road density instead of only focussing on highways or on the contrary small country roads. However, this complicates also the interpretation of the results. Small country roads have another impact than large highways and can even cause opposite effects for the same species, e.g. a goose species might profit from breeding in the high noise load zones but relative low (human presence) disturbance zones directly along a highway but might avoid zones along a small country road with more recreational use. The authors clearly are aware of this and added an analysis on a subset of minor roads.

My only major comment and question is why they did not include an analysis on a subset with roads/highways with high traffic intensity, even if the data allow only analyses for a smaller set of species. The hypothesis would be that the results show more negative impacts and larger disturbance distances in that analysis and if confirmed this would clearly add to the robustness of the study and facilitate interpretation of the results.

We agree that an analysis on both major and minor roads separately would be a great addition to the manuscript and we have now incorporated this for 16 species. In general, the effect sizes of major roads were greater, and more likely to be negative, than those for minor roads. We have discussed the results and compared them both in the manuscript and the supplementary material.

A minor general remark is that I miss some insight in what exactly the models are that were tested and what the results are. For instance, I would like to see a table showing the results of the basic GAMM models applied and not only the results for the effect of road exposure.

We have now included a table (S1.2) with the supplementary online material containing the full results of the GAMMs for all species.

Specific comments

Line 44: I suggest to add visual disturbance

Line 46: I think the evidence points out that a few kilometres is exceptional but distances up to many hundreds of meters are often mentioned, maybe better to state what is the most commonly found magnitude rather than stressing the extremes

First 2 paragraphs: maybe good to add a few remarks to the fact that road encroachment often goes together with habitat changes and also intensity of use by human activities. Often difficult to tear apart from the pure road (use) effects.

We have altered the third paragraph of the introduction to encompass these three suggestions:

"Roads are a source of noise, wildlife-vehicle collisions, chemical pollution and visual disturbance, including artificial light[17–20]. Their construction leads to fragmentation effects and changes in local habitat, and often exposes surrounding areas to further development and other human activities[21,22]. Roads have been shown to affect local populations of a range of taxa, and their impacts can extend far from the roads themselves. Studies have measured effect distances of several hundred metres, with some reporting distances of over a kilometre[20,23,24]."

Line 47-48: I think it is also important to mention that you study all paved roads and not only heavily travelled roads. It is more about the density of roads than the effect of a single (heavily used) road.

We have added a note on this towards the end of the introduction. This is also mentioned in the "Associations between road exposure and bird abundance" section.

"We use data from the extensive UK Breeding Bird Survey (BBS) to analyse populations of 75 British bird species in relation to the nation's paved road network, and to assess predictors of these patterns."

Line 70: as start of the results section I would like to see a reference to an overview of the basic results of the GAMS (in a table in supplementary section). So what are the significant variables per species?

We have now incorporated a table into the supplementary online material containing the full results of the GAMMs for all species.

Line 110. I am interested in the statistical effect of the land use intensity parameters. Were they indeed significant and do you think you corrected sufficiently for it?

We accounted for agricultural land use in three ways – using the proportion of arable land within a 5 km radius, the largest field (cropping unit) within a 5 km radius, and the farmland habitat category. 63/74 species showed a significant association with farmland habitat, 34 of which were positive and 29 were negative. Of the species for which we tested a linear association between proportion of arable land and bird abundance, 47 were significantly associated, 19 positively, 19 negatively and 9 quadratically. 8 species had significant associations with the largest cropping unit, 4 positively and 4 negatively. In general, our results for agriculture land use make sense biologically, with typical farmland birds being associated quadratically (as they tend to fair best under middling intensity), and a slight trend of specialist species being associated more negatively, and generalists more positively (correlation between prop. arable effect size and habitat specialisation = -0.3). We feel that, by using these three covariates in combination, one of which, proportion of arable land, we showed to have a high correlation with agricultural yield, we have accounted sufficiently for impacts of land use intensity.

Line 124, table 1: it seems to me that mean body mass and national population size are confounded, even if the correlation is not so high it hampers inclusion of both how do the authors interpret this? And did they explore the effect of an interaction term?

We calculated the variance inflation factors (VIFs) for all five characteristics, none of which were greater than 2. We therefore concluded that we need not be overly concerned with multicollinearity here. We chose not to incorporate an interaction term in the model used in the manuscript as we were interested in the effects of these two characteristics separately, rather than together.

Line 145: noise effects: also Halfwerk et al. (2011), disturbance effects: I think the main impacts particularly along smaller roads in the country side with recreational use (walkers and bikers) is caused by visual disturbance. There is a wide range of evidence indicating that certainly larger bodied species (e.g. waterfowl and waders) have 'flight initiation distances' up to many hundreds of meters (Livezey et al. 2016)

Line 147: likewise for open land ground nesting birds (like meadowbirds) these structures associated with roads are causing also disturbance effects (van der Vliet et al. 2010).

We have added the Halfwerk reference and included visual disturbance here.

Line 158: Collisons seems an unlikely explanation for disturbance effects up to 500 meter. The evidence points to a high impact of noise disturbance along higways in the case of songbirds. And along smaller roads the lower intensity of cars also will lead to a much lower risk of collisions.

We have changed this sentence to focus on disturbance rather than collisions.

"Smaller-bodied species and migrants may also be found in lower abundances around roads due to increased sensitivity to road-related disturbances such as noise."

Line 164: what is the likelihood that positive effects found in your analyses can be attributed to small scale subtle differences in habitats along roads or availability food/grit? Instead of only focussing on a quite formal statistical analysis I would like to have a bit more of ecological understanding/discussion of why species react like they do. This would add to the discussion/conclusion whether you think it is indeed the road/traffic that is causing effects or whether roads act as a proxy for (subtle) land use changes.

Line 165-167: Also with regards to the previous remarks I think this conclusion is not justified. Specialisation index is not automatically correcting for habitat differences linked to roads within the broad BBS habitat categories so there still is a possibility that roads are proxy for subtle habitat differences. Furthermore human disturbance is a container for all kinds of types of disturbance (noise, visual disturbance, light) so it is not clear to me how this tolerance would work. I think many of the shown associations remain a black box and therefore explanations for the observed relations remain very speculative

We have added a new section, with a more in-depth discussion of the potential role of habitat variation, and removed the speculation concerning specialisation.

"Several smaller-scale studies have shown bird abundance to increase or decrease with proximity to roads[24,37–39,72] with similar scales of change and mean effect distances to those found here[22,37,39]. Reductions in abundance may be attributed to direct mortality from collisions[19], or avoidance of areas around roads due to noise[40,67] or visual disturbance[18,28,41,68], which decrease the perceived habitat quality. This can lead not only to population reductions but also changes to population structures[69,70]. Increases in abundance could be explained by attraction to the road surface for food,

grit or heat[19,42,43], or to roadside habitat[38,44,45] and associated structures such as powerlines and fences[46].

The influence of roadside habitat is particularly difficult to quantify here as, although we incorporated habitat in our models, it was not at a high enough resolution to account for subtle changes in roadside areas. Roads can create a variety of edge habitat[38,45], which may be of benefit to some species but be avoided by others. Britain has very few areas of lowland semi-natural habitat and so road verges, which often contain hedgerows and trees, may be important for some species. In addition, many roads may have been built alongside existing edge habitat, in which some birds were perhaps already at reduced or increased abundance. However, some previous studies have controlled for habitat and still found negative effects of road traffic, including on several species in this analysis[22,37]. Most likely, our results arise from a combination of road and habitat effects, both varying in importance around different road types. That we found several species to differ in their associations with major and minor roads, with varying effect distances, suggests that different mechanisms may be of greater or lesser importance around each. In particular, our finding of several species being associated positively with minor roads and negatively with major roads, suggests high-levels of traffic may outweigh habitat benefits, even for those species that are able to cope with lower-level disturbance."

Line 169: there is some evidence for a lowered habitat quality (e.g. studies Reijnen cs).

We have now mentioned that disturbance by roads may decrease the perceived habitat quality and that this can cause population structure changes.

Line 185: is this 600 meter only for the negative impacts? Since the authors talk about conservation, declines and extinction that would justify quantifying this for only the negative impact species. Even better, also showing this for roads with heavy traffic for which maybe larger disturbance zones might be expected.

We have changed this section to focus on the distances for negative associations.

Line 188. See also Reijnen et al. (1997) for recommendations of meadow bird conservation in relations to proximity of roads.

We have now incorporated this reference with a suggestion that mitigation of noise is of particular importance.

Line 220: Would it be possible to add a figure showing the relation between k and an 'impact distance' so the reader can interpret the k-values better?

We have now produced a new figure showing the relationship between *k* and effect distance in the supplementary material (S2.4).

Line 228: but where can I see the results of the effects of these covariates?

We have now added a table in the supplementary material with the results for all covariates.

S1. Table 1.1. What is survey visit E and L? I suspect Early and Late, so please add to table heading

Thank you for spotting this error – we have now added this to the table heading.

S1. Line 238: I do not see this in table S1.1

We have now included this information in Table S1.1, thank you for alerting us to this.

I wonder how many BBS squares are located in urban areas and how the obvious higher density of roads in urban habitat is influencing the results. Have the authors tested this (e.g. by omitting urban habitats from the analyses)?

BBS squares are generally placed away from densely populated areas and in our analysis, 86% of transect sections are in areas with fewer than 1000 people per square km. Given this, we chose not to exclude urban habitat from the analysis but to account for it both in the human population covariate and the "human sites" habitat category.

Also, I am curious how many (%) transect sections are at close range of a road and even more interestingly, how many are in the close proximity of a heavily travelled road. Can you be confident that the analyses have sufficient power to detect effects. Could you show some simple statistics about that?

The majority of BBS squares are in quite close proximity to a road, giving us confidence in our results. However, the proportion of transects close to roads varies quite substantially between minor and major roads. 93% and 47% of transect sections were within 1000 m and 100 m of a minor road respectively, and 44% and 9% were within 1000 m and 100 m of a major road respectively. This is why we decided to analyse all roads together because analysing major roads on their own for species with smaller sample sizes was not possible. However, we have now added a sub-analysis of some common species using major and minor roads separately and, in the methods section regarding this, we include the above statistics on the proportions of transect sections near roads.

S2. About the habitat descriptors: what is meant by human sites, urban areas, industrial sites, railways???

We have now added more information about all of the habitat categories in the supplementary material:

"They also record the dominant habitat type in each 200-m transect section as one of nine broad classes: woodland (dominated by trees generally greater than 5 m tall); scrubland (dominated by woody shrubs or young trees less than 5 m tall); semi-natural grassland and marsh (dominated by grasses or wet communities dominated by rushes/sedges/reeds etc); heathland and bogs; farmland (enclosed fields); human sites (areas associated with people i.e. buildings, parks and gardens); water bodies (freshwater); coastal; inland rock. For full methods see BTO (2019)."

<u>Reviewer #3 (Remarks to the Author):</u>

This paper explored the effects of road density on bird populations across Great Britain. Anthropogenic influences on species habitat is a global ecological concern, and while the insights of this study are fairly local in nature, it is easy to grasp the relevance of the issues and understand how the methodology could be repeated elsewhere and for other taxonomic groups.

Simply put, I thought this was a fantastically written manuscript. The authors clearly put great care into organizing their ideas, motivating the research, explaining their methodology, and communicating their findings. The study is presented in a very accessible manner, and I think a broad group of readers will find it interesting. If all manuscripts were this well polished for submission, I would take great joy in being a more frequent reviewer.

I find no major problems with the authors' methods or findings and can wholeheartedly recommend publication of this manuscript with some minor revisions.

We thank the reviewer for this kind feedback – it was a pleasure to read, thank you.

<u>General comments:</u>

I think the manuscript would benefit from a paragraph that zooms in on an individual species or two (perhaps ones with opposite responses for narrative contrast). It would be neat to see individual species observations mapped out in relation to the road network to illustrate the types of relationships the authors describe.

We agree that this is a great addition to the manuscript and therefore have now added the following paragraph, comparing two species with opposite associations with road exposure:

*"To explain our results in more detail, we use the examples of Eurasian bullfinch Pyrrhula pyrrhula and meadow pipit Anthus pratensis, species with significant positive and negative associations with road exposure respectively. The first, Eurasian bullfinch, had a road exposure effect size of 0.21. This is the effect size where road exposure = 1, i.e. directly beside a single road (higher values of road exposure result from the cumulative effect of multiple roads). We would therefore expect Eurasian bullfinch abundance to be 23% (exp(0.21)) higher next to a road than in an area where road exposure = 0. This effect size declines with distance, becoming negligible at 290 m from a road (determined by the parameter 'k' and defined as the distance at which road exposure reaches < 0.01; **Figure 3**). Conversely, meadow pipit had a road exposure effect size of -0.24, so we predict its abundance to decrease by 21% (1-exp(-0.24)) next to a road, compared to a location with no road exposure. The maximum effect distance for meadow pipit was 350 m. These values translate to Eurasian bullfinch experiencing a 28% increase in abundance, and meadow pipit a 31% decrease in abundance, over their interquartile ranges of road exposure (**Figure 4**; **Supplementary Figure S1.1**)."*

Likewise, perhaps a bit of additional discussion about the generalizability of these results to the rest of the globe. Is Great Britain representative of broader patterns of avian diversity? Should the reader expect the same relationships to be found elsewhere? Where are the locations that are under greatest threat from road development and human growth, and how do those locations correspond with avian abundance?

We are cautious of generalising too much here, as it is hard to say how representative the patterns that we found in Britain are of those globally. We have therefore added a section in the discussion, highlighting the need for similar studies in other areas:

"Given the extent of the global road network, it is likely that our findings are not unique to Britain and so studies to test this pattern in other countries would be beneficial. Replicability of this study is dependent on wide-scale and high-resolution bird and road data but, with increasing citizen science projects worldwide, there may already be many areas in which this is possible. Furthermore, if changes in both road and bird densities were analysed over time, and areas monitored before and after road development, this could give us a stronger idea of the level of causality between the two, and an ability to predict the impact of further construction of transport infrastructure."

<u>Line comments:</u>

Lines 31 – 34: This sentence is a bit clunky as written. Additionally, the term "biodiversity" is quite broad, and can mean different things in different contexts. Perhaps reword as something like "It has been argued that differential responses in species' abilities to cope with anthropogenic disturbance are leading to reductions in beta diversity…" or "It has been argued that differential responses in species' abilities to cope with anthropogenic disturbance are leading to a homogenization of species assemblages…"

We have altered this sentence accordingly:

"It has been argued that differences in species' abilities to cope with anthropogenic disturbance are leading to simplification of species assemblages in human-disturbed environments."

Furthermore, the introductory paragraph introduces the concept of biotic homogenization, but as far as I can tell, the study does not attempt to measure or quantify homogenization in any way. In fact, this is also reflected in the manuscript title by the use of the word "communities". While the discussion comes back around and does a good job of contextualizing the results within this concept, I can't help but feel left a little wanting. It would be neat to see something that compares road density with species assemblages, for example.

We agree that homogenisation is not something we have specifically tested and so have now shifted the some of the text in the manuscript to be more focused towards the possibility of simplification of assemblages or communities, rather than biotic homogenisation per se.

Lines 38 – 39: It might be easy for the casual reader to incorrectly compare the figures in this sentence and conclude small increases (e.g., 1.3 billion to 1.5 billion). I suggest changing the latter figures to state the total expected number of road kms and vehicles rather than the increase (i.e., 95 million km of roads and 2.8 billion vehicles by 2050).

We have made the suggested change.

Line 56: There are presumably many ways in which to derive a habitat specialization index, so I think it would make more sense to say "an index of habitat specialization" rather than simply "habitat specialization index".

We have changed this throughout the manuscript.

Lines 56 – 57: The study appears to have been conducted for the entirety of the island of Great Britain, but one of the predictors is described as "UK national population size". Do these population sizes include bird populations of Northern Ireland? If not, consider renaming the predictor for clarity. (Caveat: I'm a woefully ignorant Yank when it comes to these kinds of political subtleties; if what I just said makes no sense, please feel free to disregard.)

Thank you for pointing this out – we did use population estimates for Great Britain and have now clarified this in the methods.

Line 61: Reword slightly to "…previous work has shown migrant populations to be reduced…"

We have changed this.

Line 71: Reorder to "We calculated the road exposure… …within a 5-km radius of each site using kernel density estimation." Line 72: When I read the words "composite", "density", and "paved roads" all in one sentence, my mind bizarrely tried to interpret the statement as having something to do with the physical structure of the road itself. Perhaps change to "…a combination of the geographic density and distance of all paved roads…".

We have reworded the sentence to take both comments into account:

"We calculated the road exposure of almost 20,000 BBS transect sections using the locations of all paved roads (as mapped in 2013) within a 5-km radius of the midpoint of each transect section. Within these calculations we estimated the spatial scale of the relationship between distance to road and road exposure (determined by a parameter 'k') for each species separately (see Methods for further details)."

Lines 76 – 77: What about species with fewer than 500 individual birds? Does this bias the study toward more common birds? How does the UK BBS identify individual birds over a three-year period and avoid double-counting?

We have now changed our threshold – partly for simplicity and partly because we thought it more sensible. We have analysed all species with a total annual mean count (the total of the mean count across the three-year period for each transect section) >50. This caused us to lose two species and gain three, which are now included in our results (this small change has not made any significant difference to the overall results). We feel this is a sensible cut-off as preliminary analysis indicated it to be the lowest sample size that would enable the models to run successfully. This is not only a product of the individual numbers involved, but also because many observations from rarer species come from a limited number of squares/transect sections. It would, of course, be fantastic to test all species, but we were still able to test a wide range and, given that we had some species with total mean count >20,000, we felt that 50 was a relatively low cut-off.

Line 100, Figure 2: I think a little more could be done with this figure, as it currently only really conveys a single dimension of data. For example, instead of the x symbols, you might use circles that are colored with a gradient determined by the effect sizes from Figure 1; there is some interesting reordering of species, suggesting that the largest effect sizes do not necessarily translate to the largest changes in abundance. And/or use point size to indicate total population size. It would probably be beneficial to condense the vertical space in this plot, as there is a considerable amount of white space. Also, consider removing the horizontal bars, as they don't actually contribute any new information.

We have now replaced Figure 2 so that the symbol size is determined by the effect sizes from Figure 1 and we have rearranged the figure to reduce the white space. We chose not to incorporate population size as well as this rather confused the image.

Lines 206 – 213: From the methods description, it does not appear that there is any distinction made between individual birds, and that the >500 number refers to summed population counts over the three-year study period. Given that individual birds are almost surely being counted multiple times across surveys, I would suggest rewriting this to avoid the use of "individual birds". The same suggestion applies to Lines 76 – 77.

We have adjusted this section in accordance with our earlier answer regarding our change in threshold.

Additional changes

As part of these revisions, we went through all our data and code to make sure it was correct and easily clearly replicable. In doing so, we found a small inconsistency in the inclusion of minor habitat categories for some species. We have now fixed this and rerun the models. It made almost no difference to the individual results, but it did tighten up some of the confidence intervals. In response to one reviewer's comment we also changed our threshold for inclusion of species, resulting in us adding one bird species to the analysis. These two changes caused migratory tendency to become significant in the generalised estimating equation, along with national population size and body mass. We therefore updated the text and figures in the manuscript to accommodate this.

REVIEWERS' COMMENTS:

Reviewer #2 (Remarks to the Author):

Comments on the revised version of the manuscript 'Roads as a contributor to landscape-scale variation in bird communities', Cooke et al.

I am very pleased with the current version of the manuscript. Although the earlier version already showed a very high quality it is nice to see that the authors were able to clearly improve the impact and robustness of their results. I am particularly pleased that they decided to follow my suggestion to include an analysis for major and minor roads separately. The results are very convincing. Major roads clearly show more negative results and the few exceptions (Jackdaw and Rook) are indeed well-known species that even prefer to stay and/or breed along heavily travelled roads. I welcome the additional information in the method and result section about the analyses. The way the authors dealt with the remarks made by the reviewers is very convincing and satisfactory.

Reviewer #3 (Remarks to the Author):

Summary:

As with the first draft, I thought this was a fantastically written manuscript. The authors did an excellent job of responding to the reviewers' comments, and their additional efforts have improved the presentation of their findings considerably. I find no major problems with their manuscript, and the only comments I have to provide are considerably minor, and either pertain to figure improvements or suggestions of grammar. I assume that the authors will respond to my suggestions appropriately, and I do not need to see another draft to recommend this manuscript for publication.

Line comments:

Line 127: Axis label should be "Distance from road (km)" not "Distance from road/km".

Line 135: Axis labels should include units of measurement.

Line 154: This figure would benefit greatly from a legend.

Lines 167-170: The previous draft of this manuscript mentioned using taxonomic family as a grouping factor to address phylogenetic relatedness, but this approach is no longer described in the methods (Lines 340-352). If this portion of the analysis was removed, then these sentences should also be removed.

Line 181: Since all three panels share the same y-axis, I would recommend reorganizing this figure into a 1x3 layout, with y-axis labels and numbers shown only once on the first panel, and simple tick marks on the other two. This figure would likewise benefit from a legend.

Line 188: Consider rewording "so are those that had the strongest influence" to "and thus had the strongest influence".

Lines 214-215: Remove "That" at the beginning of the sentence and replace "suggests" with "which suggests".

Lines 217-218: Replace "major roads, suggests high-levels of traffic" with "major roads suggests that high levels of traffic". Change "several species being associated positively" to "several species positively associated".

Lines 244-251: I really like the addition of these discussion points, not only addressing the generalizability of the results but how future study designs might leverage these results to gain better insight. Nicely stated!

Lines 277-279: This says that species with total mean annual count of <100 were removed, but the reviewer response states that species with total mean annual count >50 were kept. This is a discrepancy, and I'm not sure which is correct, but the authors should double-check this and correct as needed.

Cooke et al. Roads as a contributor to landscape-scale variation in bird communities

Response to reviewers.

Reviewer #3 (Remarks to the Author):

Summary:

As with the first draft, I thought this was a fantastically written manuscript. The authors did an excellent job of responding to the reviewers' comments, and their additional efforts have improved the presentation of their findings considerably. I find no major problems with their manuscript, and the only comments I have to provide are considerably minor, and either pertain to figure improvements or suggestions of grammar. I assume that the authors will respond to my suggestions appropriately, and I do not need to see another draft to recommend this manuscript for publication.

Line comments:

Line 127: Axis label should be "Distance from road (km)" not "Distance from road/km".

We have changed this.

Line 135: Axis labels should include units of measurement.

Road exposure does not have a unit of measurement as such and we have made clear in the figure legend that "estimated bird abundance" refers to the number of birds around a single transect section.

"Figure 4. Estimated abundance of two species across the full range of road exposure recorded for each. Bird abundance refers to the number of birds within 100 m of a 200-m BBS transect section. The 0.25 and 0.75 quartiles of road exposure for each species are indicated by the vertical lines and 95% prediction intervals by the shaded areas. These graphs are available for all species in Supplementary Figure 1."

Line 154: This figure would benefit greatly from a legend.

We have not included legends on the figures but retained the text description of the colour coding in the separate figure legend, in keeping with the formatting requirements of the journal.

Lines 167-170: The previous draft of this manuscript mentioned using taxonomic family as a grouping factor to address phylogenetic relatedness, but this approach is no longer described in the methods (Lines 340-352). If this portion of the analysis was removed, then these sentences should also be removed.

We have reinserted the missing sentence in the methods.

Line 181: Since all three panels share the same y-axis, I would recommend reorganizing this figure into a 1x3 layout, with y-axis labels and numbers shown only once on the first panel, and simple tick marks on the other two. This figure would likewise benefit from a legend.

We have changed this figure to a 1x3 layout and also made similar changes to Figure 4. As above, we have not included legends on the figures but retained the text description of the colour coding in the separate figure legend, in keeping with the formatting requirements of the journal.

Line 188: Consider rewording "so are those that had the strongest influence" to "and thus had the strongest influence".

We have done this.

Lines 214-215: Remove “That” at the beginning of the sentence and replace “suggests” with “which suggests”.

We have done this.

Lines 217-218: Replace “major roads, suggests high-levels of traffic” with “major roads suggests that high levels of traffic”. Change “several species being associated positively” to “several species positively associated”.

We have done this.

Lines 244-251: I really like the addition of these discussion points, not only addressing the generalizability of the results but how future study designs might leverage these results to gain better insight. Nicely stated!

Lines 277-279: This says that species with total mean annual count of <100 were removed, but the reviewer response states that species with total mean annual count >50 were kept. This is a discrepancy, and I’m not sure which is correct, but the authors should double-check this and correct as needed.

The 100 in the paper was correct and has been retained.

<u>Additional changes</u>

We have added one figure to the manuscript, in the Discussion, to show the areas of Great Britain and its terrestrial protected areas that lie within 700 m of a road.

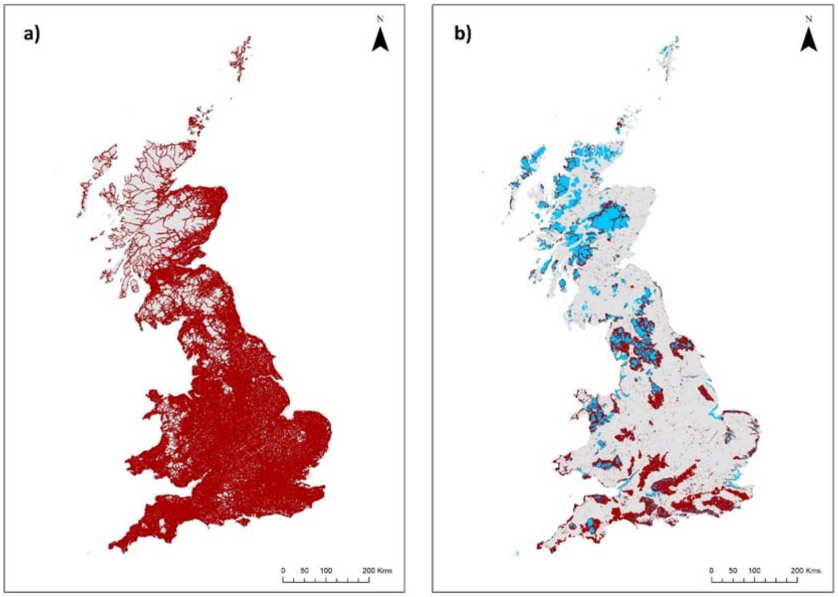

Figure 7. Areas of **a)** Great Britain and **b)** terrestrial protected areas that lie within 700 m of a road. Blue represents terrestrial protected areas and red represents areas of **a)** Great Britain and **b)** terrestrial protected areas within the mean effect distance, 700 m, of associations between roads and bird abundance variation.
